# Enhancement of Cerium Sorption onto Urea-Functionalized Magnetite Chitosan Microparticles by Sorbent Sulfonation—Application to Ore Leachate

**DOI:** 10.3390/molecules27217562

**Published:** 2022-11-04

**Authors:** Mohammed F. Hamza, Eric Guibal, Adel A.-H. Abdel-Rahman, Marwa Salem, Mahmoud S. Khalafalla, Yuezhou Wei, Xiangbiao Yin

**Affiliations:** 1 School of Nuclear Science and Technology, University of South China, Hengyang 421001, China; 2 Nuclear Materials Authority, P.O. Box 530, El-Maadi, Cairo 4710030, Egypt; 3 Polymers Composites and Hybrids (PCH), IMT Mines Alès, F-30360 Alès, France; 4 Chemistry Department, Faculty of Science, Menofia University, Shebin El-Kom 32511, Egypt; 5 School of Nuclear Science and Engineering, Shanghai Jiao Tong University, Shanghai 200240, China

**Keywords:** bi-functionalization, magnetic chitosan, cerium recovery, sorption isotherm, uptake kinetics, metal desorption and sorbent recycling, cerium recovery from processed leachate

## Abstract

The recovery of strategic metals such as rare earth elements (REEs) requires the development of new sorbents with high sorption capacities and selectivity. The bi-functionality of sorbents showed a remarkable capacity for the enhancement of binding properties. This work compares the sorption properties of magnetic chitosan (MC, prepared by dispersion of hydrothermally precipitated magnetite microparticles (synthesized through Fe(II)/Fe(III) precursors) into chitosan solution and crosslinking with glutaraldehyde) with those of the urea derivative (MC-UR) and its sulfonated derivative (MC-UR/S) for cerium (as an example of REEs). The sorbents were characterized by FTIR, TGA, elemental analysis, SEM-EDX, TEM, VSM, and titration. In a second step, the effect of pH (optimum at pH 5), the uptake kinetics (fitted by the pseudo-first-order rate equation), the sorption isotherms (modeled by the Langmuir equation) are investigated. The successive modifications of magnetic chitosan increases the maximum sorption capacity from 0.28 to 0.845 and 1.25 mmol Ce g^−1^ (MC, MC-UR, and MC-UR/S, respectively). The bi-functionalization strongly increases the selectivity of the sorbent for Ce(III) through multi-component equimolar solutions (especially at pH 4). The functionalization notably increases the stability at recycling (for at least 5 cycles), using 0.2 M HCl for the complete desorption of cerium from the loaded sorbent. The bi-functionalized sorbent was successfully tested for the recovery of cerium from pre-treated acidic leachates, recovered from low-grade cerium-bearing Egyptian ore.

## 1. Introduction

Cerium is one of the light rare earth elements (LREEs). It is mainly used in industry as a non-toxic pigment in the manufacturing of flat-screen monitors, low-energy light lamps, and floodlights, and in the synthesis of catalysts (under oxide form, in applications such as auto-cleaning systems, catalytic converters for diesel fuel, and so on). The relative supply risk of cerium is ranked highly (9.5 on a 10-point scale). Indeed, most of its production (about 97%) is located in three countries (i.e., China, Russia, and Malaysia), though the reserves are more dispersed (50% of reserves being located in China, CIS countries, and USA) [1]. These characteristics (commune with the complete family of REEs), combined with the difficult substitutability of this metal, and the limited rate of recycling (evaluated as close to 10%) may explain the strong attention paid to the development of processes focused on the recovery of these strategic REEs (including cerium) from alternative resources, such as acid mine drainage (AMD) [2], marginal ores [3] and tailings [4,5,6], urban mines [7], and waste electric and electronic equipment (WEEEs) [8,9].

The treatment of solid wastes and ores may involve (after dismantling and/or grinding) gravimetric and magnetic separations. Roasting [10] and leaching [11] constitute the processes most frequently applied to these solid wastes via the pyro- and hydro-branches of extractive metallurgy, respectively [12]. The leaching steps may include acidic [13], alkaline [14], or bioleaching solutions [6,15]. Simple precipitation techniques are poorly selective for the treatment of leachates [16,17], and it is generally necessary to couple selective precipitation steps [13,18,19] with complementary separation processes, such as solvent extraction [20,21,22] or sorption processes [23,24]. Sorption processes are usually preferred to solvent extraction for the treatment of low-concentration effluents. A wide range of materials have been reported for cerium or REE sorption, including biosorbents [25,26,27,28,29,30], carbon-based supports [24,31,32], inorganic sorbents [33,34], and chelating and ionic-exchange resins [35,36,37,38,39,40,41].

The commercial resins applied to the recovery of rare earth elements may bear a wide variety of functional groups, such as sulfonic (Amberlite 200C H, or Lewatit Monoplus SP112, Lewatit MDS 200H) or sulfonate (Amberlite 200C H), picolylamine (Dowex M 4195, Dowex XUS43605) [36,41,42,43], sulfonic/phosphonic (Purolite S957) [44], iminodiacetic (Amberlite IRC748, Purolite S930, or Lewatit Monoplus TP208, Lewatit TP207), aminophosphonic (Amberlite IRC747, Purolite S940, Purolite S950) [41,45], or diphosphonic/sulfonic/carboxylic groups (Diphonix) [46]. By comparison with these commercially available resins, numerous investigations have focused on the grafting of supports with similar functional groups, such as aminophosphonic onto activated carbon [47], an algal/PEI support decorated with sulfonic groups [48] or phosphonate moieties [49], phosphonate onto metal organic framework [50], and diethylenetriamine incorporated onto magnetic chitosan microparticles [51]. Callura et al. [52] compared a series of aminated PS-DVB beads grafted with a wide series of functional groups (phosphonoacetic acid, phosphonomethylglycine, or diethylenetriaminepentaacetic acid di-anhydride) for the sorption of Nd(III), Gd(III), and Ho(III).

## 2. Selected Strategy for Sorbent Synthesis—Rationales

Chitosan (aminopolysaccharide) has been widely investigated for metal sorption due to its hydrophilic character (associated with numerous hydroxyl groups) and the high reactivity of the amine groups [53,54,55]. Chitosan is characterized by its unique cationic behavior among carbohydrates. The protonation of the amine groups (at a pH below pK_a_, in the range 6.2–6.7) has been used for binding metal anions and anionic dyes. On the other hand, in near-neutral solutions, free amine groups can chelate metal cations. These hydroxyl and amine groups have been used for preparing numerous derivatives through the grafting of functional groups [56,57,58], or physical modifications (for preparing beads, fibers, membranes, foams, etc.) [59,60].

One of the main drawbacks affecting chitosan’s application in water treatment consists of the poor porosity and the residual crystallinity of the biopolymer that may explain its slow mass transfer properties. This drawback can be overcome by using different strategies: (a) coating the biopolymer onto a highly porous support [61,62], (b) re-conditioning the biopolymer (the dissolving of chitosan reduces its residual crystallinity and the gelling or neutralization step allows for the expansion of the hydrogel network, pending a strict control of the final drying step) [63], and (c) reducing the size of the sorbent particles [64].

Reducing the size of sorbent particles decreases the diffusion path and, consequently, the time required to reach the equilibrium, although this process necessitates the performance a difficult solid/liquid separation at the end of the operative steps. This disadvantage can be minimized through the incorporation of magnetic nanoparticles that facilitate the magnetic separation of the sorbent at the end of the process [65,66]. In some cases, the preparation of magnetic sorbents proceeds through the simultaneous synthesis of magnetite nanoparticles and precipitation of chitosan (first, by mixing the Fe(III) and Fe(II) precursors with chitosan before processing the hydrothermal precipitation at a controlled pH). Herein, the magnetite NPs were pre-synthesized before being embedded into chitosan hydrogel.

The solubility of chitosan in acidic solutions (with the remarkable exception of sulfuric acid) usually requires the crosslinking of the biopolymer to reinforce the sorbent stability when acidic conditions are involved in the lifecycle of the sorbent (especially for metal desorption and sorbent recycling, [67]). In many cases, the crosslinking step results in a loss of sorption properties because amine groups are frequently involved in the crosslinks (typically, this is the case for glutaraldehyde crosslinker). When chelation mechanisms constitute the main binding mechanisms, the sorption capacity dramatically falls, contrary to ion exchange mechanisms, which are less affected. The sorption properties of raw chitosan for rare earths are relatively limited (see below, MC sorbent: crosslinked magnetic sorbent). Therefore, the functionalization is necessary for improving the potential of magnetic chitosan.

Table S1 (Section SA in Supplementary Information) reports the maximum sorption capacities of a series of functionalized sorbents for REE(III) metal ions (some of which concern magnetic chitosan). It is remarkable to note that the beneficial effect of grafting new functional groups onto supports strongly depends on the metal. For example, in the case of the algal/PEI sorbent, the sulfonation of the hydrogel contributes to the material’s strong sorption capacity for scandium (i.e., 3.16 mmol Sc g^−1^), while for cerium and holmium, the sorption levels remain much lower (i.e., 0.71–0.61 mmol g^−1^) [48]. The bi-functionalization of a support may increase the sorption performance, as in the case of La(III) and Ce(III) with alginate sorbent functionalized with poly-glutamate [68]. The bi-functionalized sorbents that are most frequently reported for their REE sorption capacities bear amine and carboxylic groups. The literature is less abundant concerning the sulfonic/amine multifunctional materials. The current work investigated the functionalization of magnetic chitosan particles by first grafting urea (mediated by formaldehyde) to produce an amine-enriched sorbent (which is stabilized with glutaraldehyde crosslinking). In a second step, some of these amine groups were grafted with sulfonated groups (through the use of a synthesized sulfonated agent (N(SO_3_Na)_3_).

The comparison of the sorption performances for Ce(III) sorption offers insight into the impacts of mono- and bi-functionalization (amine and sulfonic groups) on the enhancement of the binding properties and the selectivity in sorption. The sorbents were characterized using different analytical facilities (a detailed discussion appears in Supplementary Information). The sorption properties are investigated within the scope of the pH effect, uptake kinetics and sorption isotherms (with conventional models), selectivity properties (at different pH values with multi-component solutions), desorption performance, and stability in recycling. In the last part of the study, the bifunctional sorbent was applied to metal recovery from the raffinate of the acidic leachate of Egyptian ore. The raw leachates were pre-treated with Amberlite IRA-400 (for Fe(III) removal) and Dowex 50 X8 (for the extraction of REEs). MC-UR/S was tested for the valorization of the residues. These tests allowed us to evaluate the tested materials’ potential for the recovery of Ce(III) and REEs from complex solutions.

## 3. Results and Discussion

### 3.1. Characterization of the Sorbents

The full characterization of the sorbents is detailed in Section SB (SI). Herein, the main characteristics are summarized. The particles are characterized as elongated irregular objects with rounded edges (Figure S1 Supplementary Material). The average size of the microparticles of MC-UR/S is 6 ± 4 µm. The embedded nanoparticles (NPs) of magnetite are visible in the TEM images (Figure S2, with a size of around 5–7 nm). The magnetic properties of MC-UR/S were quantified by vibrating sample magnetometry to 18.7 emu g^−1^, with limited coercivity and remanence. The particles are superparamagnetic (Figure S3). This low value can be explained by the effect of the polymer coating of the magnetic NPs and the low fraction of magnetite in the composite. Indeed, the TGA analysis shows that the residual weight (after reaching 800 °C) shows a progressive decrease with the functionalization of the MC material, reaching 56.0%, 45.2% for MC-UR, and decreasing to 26.4% for the sulfonated sorbent (Figure S4). In addition, the TGA profiles show three main transitions corresponding to water release, followed by the depolymerization of chitosan, together with the degradation of the end products (urea and sulfonated ends), before recording the degradation of the char. Intermediary sub-waves are also observed (and confirmed by the DrTG profiles, Figure S5), which are probably associated with the degradation of functionalizing moieties.

**Figure 1:**
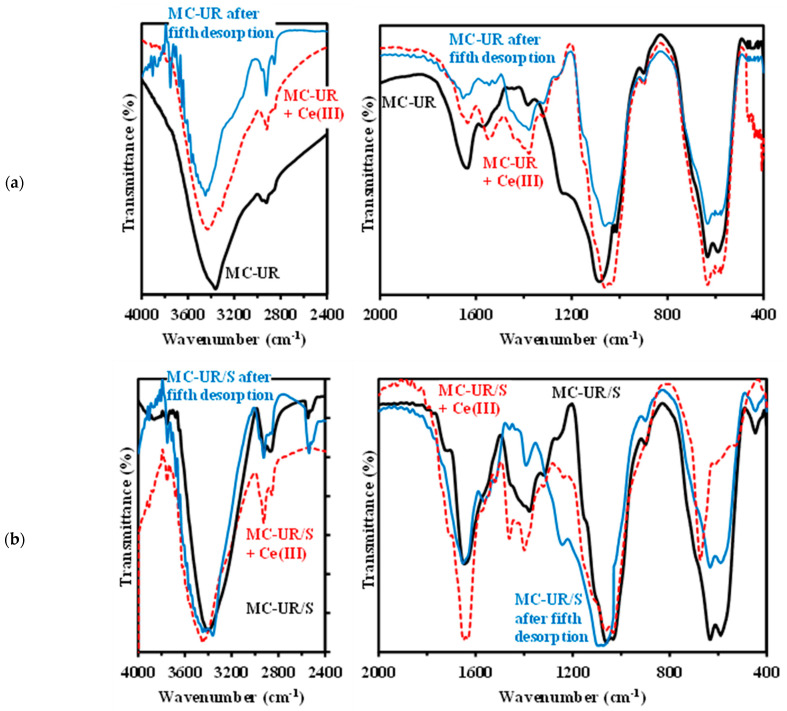
FTIR spectra—(**a**) MC-UR and (**b**) MC-UR/S, raw, after Ce(III) sorption, and after 5th desorption.

The analysis of the FTIR spectra for MC-UR and MC-UR/S at different stages of use (raw, after Ce(III) sorption, and after five cycles of re-use) shows, firstly, information regarding the presence of reactive groups (amine, amide, carbonyl, and sulfonate) and their interactions with cerium. Several characteristic bands of these functional groups are shifted after metal binding and desorption (Figure 1, Table S2a).

However, these changes may result not only from the interaction of the sorbent with Ce(III). Indeed, the change in the environmental conditions, such as the pH for processing the sorption (i.e., at pH 5) or the drastic acidic solution for processing the metal desorption (i.e., 0.2 M HCl), may also affect the chemical state of these reactive groups (and their relevant vibrations in the FTIR spectra).

In Section SB, the comparison of the spectra with those of the sorbents conditioned at pH 5 and exposed to 0.2 M HCl solutions is presented in Figures S6–S9 and Table S2b,c. The discussion of the relevant differences shows specific modifications in the large band at 3430–3370 cm^−1^, which is assigned to the overlapping of ν(OH) and ν(NH) vibrations, at ≈1450 and ≈1375 cm^−1^ (amide and amine groups, respectively), and at ≈1250–1050 cm^−1^, associated with ν(C-O) and δ(OH)) for MC-UR and with ν(S-O) for MC-UR/S. The investigation of the effects of metal desorption and sorbent recycling also shows substantial changes (appearance/disappearance of the signals and shifts in their characteristic wavenumbers, Figures S7 and S9, Table S2c). Despite these changes in the FTIR spectra, the full desorption of the metal and the weak loss in the sorption capacity (see Section 3.2.5) mean that these chemical modifications hardly affect their sorption performances.

The elemental analysis of the sorbent identified the iron content as being in the range 30.7–32.5% (Table S3), meaning a higher value than that deduced from the TGA analysis (at least for MC-UR/S). The successful amination (urea grafting, for MC-UR) and sulfonation (for MC-UR/S) are also demonstrated by the increase in the nitrogen content (around 6.5%, *w*/*w*; ≈4.6 mmol N g^−1^) and the appearance of the S element (around 3.3%, *w*/*w*; ≈1 mmol S g^−1^). The pH_PZC_ value of MC (close to 6.2) was increased to ≈6.6 after the grafting of the urea (weakly basic groups), while the subsequent sulfonation of the derivative caused the pH_PZC_ to shift toward lower values (i.e., 5.4) (Figure S10). These shifts allow us to anticipate differences in the pH profiles for Ce(III) sorption, correlated with the deprotonation of the functional groups.

### 3.2. Sorption Properties

#### 3.2.1. Effect of pH

**Figure 2:**
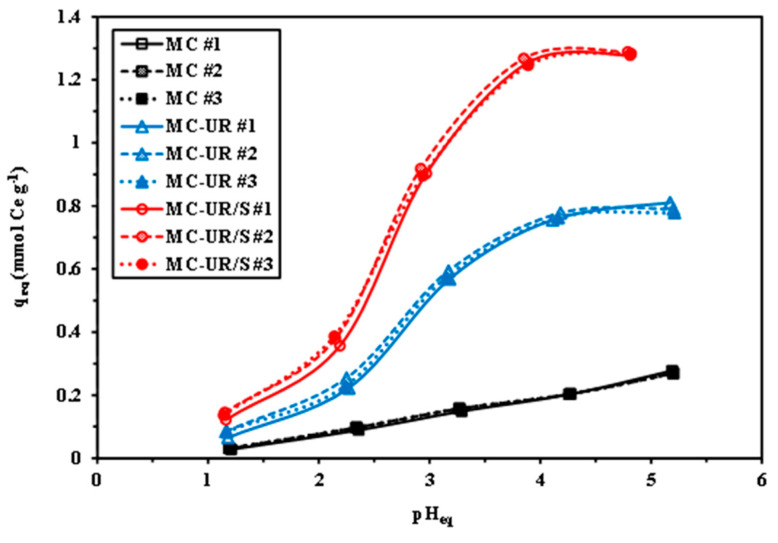
Influence of pH on Ce(III) sorption using MC, MC-UR, and MC-UR/S sorbents (sorbent dose, SD: 400 mg L^−1^; C_0_: 0.366 mmol Ce L^−1^; time: 48 h; v: 210 rpm; T: 22 ± 1 °C).

Figure 2 compares the effects of the pH_eq_ on Ce(III) sorption using MC, MC-UR, and MC-UR/S (the triplicated experiments showed a good reproducibility). These results show that the sorption capacity increases with the pH, almost linearly for MC and more marked for MC-UR and MC-UR/S.

For the functionalized sorbents, the sorption slightly increases between 1.2 and 1.5 (with sorption capacities below 0.2 and 0.5 mmol Ce g^−1^). A steep increase is observed up to pH_eq_ 4–4.3 (q_eq_ tends toward 0.8 and 1.3 mmol Ce g^−1^, for MC-UR and MC-UR/S, respectively). Above pH_eq_ 4.5, the sorption capacities stabilize. In order to avoid any risk of precipitation (especially at high concentrations, such as those used for sorption isotherms), a pH above 6 was not tested, and further experiments were performed at pH_0_: 5.

Figure S11 shows the distribution of Ce(III) species as a function of the pH (under experimental conditions selected for the study of the pH effect). At pH > 2, cerium is present only as free Ce^3+^ species. In acidic solutions, the protonation of reactive groups (as protonated amine groups, sulfonic acid moieties) involves the repulsion of Ce^3+^ cations (and CeCl^2+^ at pH < 2) and limited sorption capacities. As the pH increases, this repulsion decreases, and the sorption steeply increases, with this effect being especially notable below pH_eq_ 4–4.5. The sulfonated derivative shows a higher sorption capacity due to the increased density of the reactive groups (amino and sulfonate groups).

Figure S12 shows that for MC and MC-UR, the sorption of Ce(III) is systematically followed by a weak increase in the pH (by less than 0.3 units). On the other hand, for MC-UR/S, consistent with its lower pH_PZC_ value, the Ce(III) sorption slightly increases the pH below pH 3, while above the equilibrium, the pH tends to decrease.

In ion exchange processes, the slope of the log_10_ plot of the distribution ratio D (D: q_eq_/C_eq_, L g^−1^) vs. pH gives the ionic charge of the exchanged metal ion [69]. Figure S13 shows that the linear plot for the functionalized sorbent is close to 0.5. This value is not consistent with the theoretical interpretation described here, meaning that the binding mechanism is not purely ion exchange.

#### 3.2.2. Uptake Kinetics

**Figure 3:**
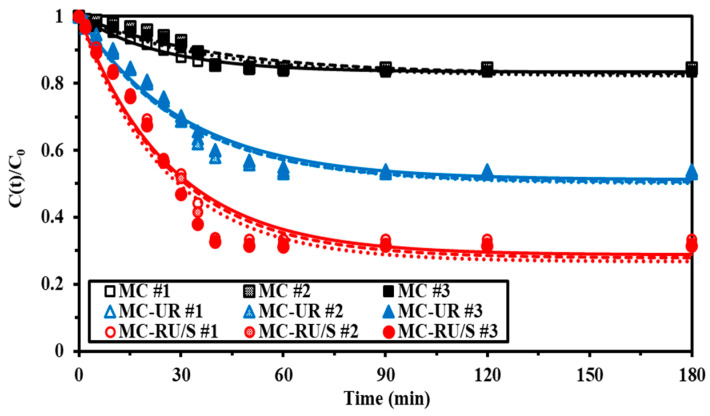
Uptake kinetics for Ce(III) sorption using the MC, MC-UR, and MC-UR/S sorbents (pH_0_ 5; C_0_: 0.732 mmol Ce L^−1^; SD: 400 mg L^−1^; v: 210 rpm; T: 22 ± 1 °C).

The uptake kinetics are remarkably reproducible (note fine superposition of the triplicated curves, Figure 3). The equilibrium is reached within 30–60 min. The micron size of the sorbent particles may explain these fast equilibria. However, the t_50_ (time required to reach 50% of the total sorption) is close to 15 min. The sorption kinetics may be controlled by the proper reaction rate (which is frequently associated with a pseudo-first- or pseudo-second-order rate equation, PFORE or PSORE, Table S4) and/or resistance to diffusion (external and intraparticle diffusions). The lines in Figure 3 show the PFORE fits of the kinetic profiles. The model roughly fits the experimental data. This is consistent with the comparison of the statistical criteria (i.e., R^2^ and AIC) for PFORE, PSORE, and RIDE (resistance to intraparticle diffusion, according to the Crank equation, Table S12a). The quality of the fitting is demonstrated by the higher values of R^2^ and lower values of AIC (the difference is significant when ǀ∆AICǀ > 2): PFORE > PSORE > RIDE.

The PFORE slightly overestimates the q_eq_ values (by 5–10%), which increase with the successive functionalizations: 0.287 ± 0.012, 0.844 ± 0.010, and 1.233 ± 0.021 mmol Ce g^−1^ (for the experimental values; 0.310 ± 0.017, 0.898 ± 0.008, and 1.310 ± 0.024 mmol Ce g^−1^ for the calculated values). The apparent rate coefficients (k_1_ × 10^2^ min^−1^) are of the same order for the three sorbents (though, in the case of MC, a greater dispersion of the data is observed): 3.11 ± 0.73, 3.26 ± 0.05, and 3.81 ± 0.08 min^−1^, for MC, MC-UR, and MC-UR/S, respectively. These values are of the same order of magnitude as the apparent rate coefficients reported by Kołodyńska et al. [31] (0.8–1.4 10^−2^ min^−1^) for Nd(III), Ce(III), and La(III) using composite biochar.

Sometimes, the fit of the kinetic profiles with the PFORE and/or PSORE is associated with the prevalence of physi- or chemisorption. However, Simonin [70] and Hubbe et al. [71] demonstrated the difficulty of correlating the controlling mechanism with the mathematical fit because of the inappropriate selection of the experimental conditions. Herein, the apparent rate coefficients of PFORE are simply discussed as a tool for objectively comparing the systems. The RIDE gives less accurate fits of the kinetic profiles, but the effective diffusivity coefficients deduced from these curves can be compared with the self-diffusivity of Ce(III) in water (i.e., D_0_(Ce^3+^): 3.72 × 10^−8^ m^2^ min^−1^} [72]). The values are several orders of magnitude lower than D_0_(Ce^3+^), meaning that the resistance to intraparticle diffusion contributes to the overall control of the uptake kinetics.

#### 3.2.3. Sorption Isotherms

**Figure 4:**
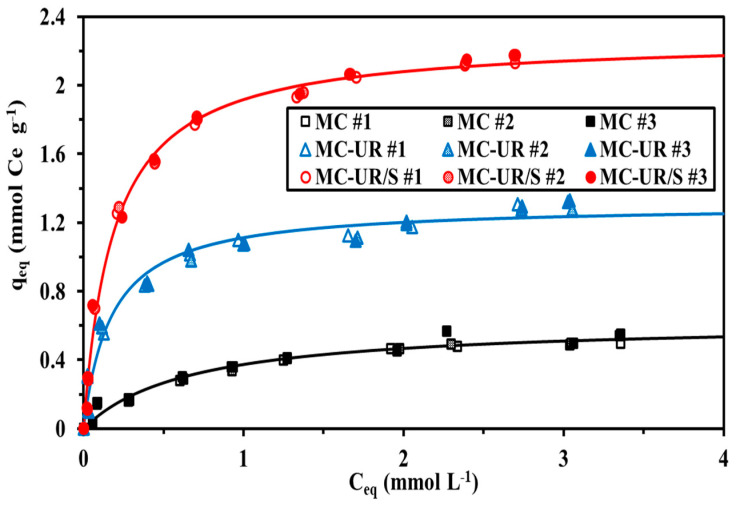
Ce(III) sorption isotherms for MC, MC-UR, and MC-UR/S sorbents at pH_0_ 5—modeled with the Langmuir equation (sorbent dose, SD: 400 mg L^−1^; C_0_: 0.007–3.57 mmol Ce L^−1^; time: 48 h; v: 210 rpm; T: 22 ± 1 °C).

The sorption isotherms (i.e., plots of the sorption capacity vs. equilibrium concentration at fixed values of the pH and temperature) are represented in Figure 4 (herein, pH_0_ 5; T: 22 ± 1 °C). The superposition of the triplicated curves confirm the good reproducibility of the sorption experiments. The sorption isotherms are characterized by a pseudo-asymptotic shape. The asymptote is correlated with the maximum sorption capacity (or sorption capacity at the saturation of the monolayer in the Langmuir model). This maximum sorption capacity (i.e., q_m,exp_), consistent with previous observations, increases with the successive functionalizations of the support: 0.549, 1.328, and 2.176 mmol Ce g^−1^ (for MC, MC-UR, and MC-UR/S, respectively). The sorption capacity increases almost linearly with the number of potential reactive groups on the sorbent (i.e., Σ = n(N) + n(S), in molar units g^−1^): q_m_ = 0.647 Σ − 1.558.

The sorption isotherms were fitted using the Langmuir, Freundlich, Sips, Temkin and Dubinin–Radushkevich (D-R) equations (Table S12b). The Freundlich equation is an empirical model based on the concept of heterogeneous sorption, with possible interactions of the sorbent molecules. Its mathematical expression (i.e., power-like function) is not consistent with the asymptotic trend of the experimental profiles. Logically, this model gives the poorest correlation between the experimental and fitted curves. The Langmuir equation accounts for homogeneous sorption without interactions with the sorbed molecules and with a similar distribution and binding energies. The Sips equation is a combination of the Langmuir and Freundlich equations. Introducing a third adjustable parameter facilitates the fitting of the experimental data (with a weaker physical significance). The adaptations of the Temkin and the Dubinin–Raduskevich equations to liquid/solid systems are strongly debated in terms of both the equations and interpretations [73,74]. Therefore, a case where these equations closely fit the experimental profiles must be considered very carefully. Tables S5–S7 summarize the parameters and the statistical criteria for the triplicates (and for the composite isotherm obtained by combining the triplicates) for the five models and the three sorbents. In the case of the MC sorbent, the Langmuir and the Sips equations gave comparable qualitative fittings of the experimental curves. However, the fitting advantage of the Langmuir equation consists of the closer values for q_m,L_ with q_m,exp_ (compared with q_m,S_). 

The Temkin equation fits the isotherms for MC-UR slightly better (preferentially to Langmuir ≈ Sips). It is noteworthy that the energetic term (b_T_) decreases with the functionalization of the material (according ≈21 > ≈11 > ≈5 J kg mmol^−2^). In the case of MC-UR/S, the Langmuir equation is again the most appropriate for describing the Ce(III) isotherm (Langmuir > Sips ≈ Temkin). Despite the less accurate fit of the isotherm with the D-R equation, the characteristic free energy of the adsorption (E_DR_) can be used to analyze and compare the sorption behavior of the three sorbents. The EDR for MC is a little lower (i.e., around 3.6 kJ mol^−1^) than the values obtained for the functionalized sorbents (i.e., around 6 kJ mol^−1^).

The Langmuir equation shows a better fit for all of the three systems, making the comparison of the model parameters easier (in addition to the more accurate determination of the sorption capacity at saturation). The q_m,L_ values increase from 0.626 ± 0.028 mmol Ce g^−1^ to 1.305 ± 0.022 and 2.275 ± 0.017 mmol Ce g^−1^ for MC-UR and MC-UR/S, respectively. The affinity coefficient (i.e., b_L_) for MC (≈1.506 ± 0.156 L mmol^−1^) is about 3 times lower than the values obtained for the functionalized sorbents (≈5.817 ± 0.720 and ≈5.379 ± 0.092 L mmol^−1^ for MC-UR and MC-UR/S, respectively). The grafting of the urea onto MC doubles the maximum sorption capacity and almost quadruples the affinity of the sorbent for Ce(III). After sulfonation, the affinity slightly decreases (by 8%), while the maximum sorption capacity increases by 74% (proportionally to the change in the total number of reactive groups).

MC-UR/S represents a good compromise in terms of the enhancement of the Ce(III) sorption properties. These properties can be compared to those of alternative sorbents. Table S8 shows that MC-U/S is comparable to the best sorbents reported in the literature. The sorbents with the higher sorption capacities (i.e., higher than 2 mmol Ce g^−1^) are based on magnetic cellulose [75], a carboxymethylcellulose highly opened monolith [76], a polystyrene/poly(hydroxamic acid) copolymer sorbent [77], or a HKUST-1 metal–organic foam [78]. It is noteworthy that MC-UR/S is characterized by one of the highest affinity coefficients (with the remarkable exception of HKUST-1-MOF). Combining these remarkable characteristics, the fast kinetics, and the easy synthesis procedure, MC-UR/S is one of the most promising sorbents described in the literature for Ce(III) recovery.

**Scheme 1:**
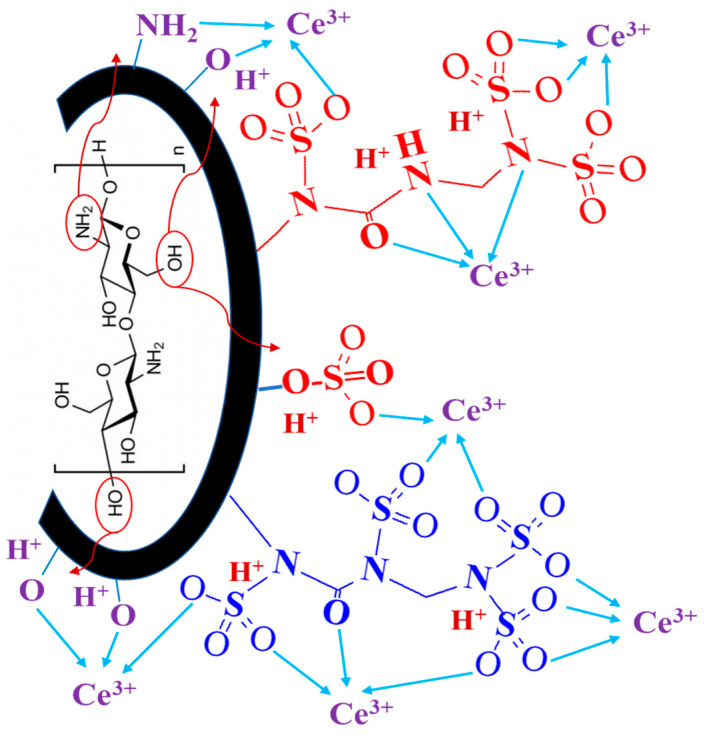
Suggested mechanisms of Ce(III) binding onto MC-UR/S.

Based on the effect of the pH and the FTIR analysis, it is possible to suggest a series of mechanisms involved in the binding of Ce(III) onto MC-UR/S (Scheme 1). In addition to the modes of interaction of lanthanides with sulfonate groups (as identified by Kołodyńska et al. [42] for the Lewatit Monoplus SP112 macroporous cation exchanger), the amine/amide groups present on urea moieties may also contribute to metal binding. These sorption mechanisms may be affected by the reported oxidation of Ce(III) to Ce(IV). However, XPS analyses were not performed to verify the natural occurrence of oxidation mechanisms under the selected experimental conditions.

#### 3.2.4. Sorption Selectivity

The selectivity of the sorbent is a key parameter in the design of new sorbents. To evaluate the preference of the sorbents for the target metals (i.e., Ce(III) and, more generally, REEs), the sorption properties of MC-UR and MC-UR/S were tested using equimolar multicomponent solutions, including Ca(II), Mg(II) (among the alkali-earth elements), Zn(II), Al(III), and Fe(III), as heavy metal ions (including trivalent cations), and Nd(III) as a competitor REE. Figure S14 shows the log_10_ plot of the distribution coefficient vs. the equilibrium pH for the two functionalized sorbents. Firstly, we can observe that the distribution ratio is a little higher for the sulfonated sorbent. The bi-functionalization allows for the increase in the selectivity for the REEs (herein, Nd(III) and Ce(III)), especially at the highest pH values (at pH_eq_: 3.7–4.4).

**Figure 5:**
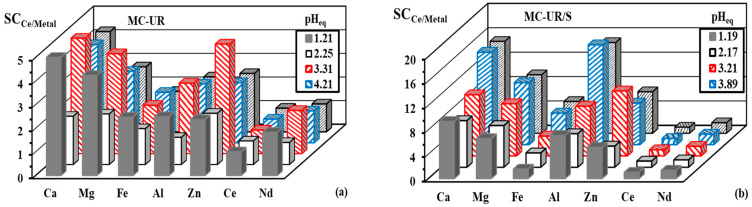
Selectivity tests for Ce(III) sorption using the MC-UR (**a**) and MC-UR/S (**b**) sorbents and the multicomponent equimolar solutions—effect of the pH_eq_ on SC_Ce/metal_ (SD: 1 g L^−1^; C_0_: ≈1 mmol L^−1^; time: 10 h; v: 210 rpm; T: 22 ± 1 °C).

In Figure 5, the selectivity coefficients SC_Ce/metal_ are compared for the different competitor metals for the two functionalized sorbents, while Figure S15 visualizes the plots for SC_Nd/metal_. The improvement in the selectivity by sulfonation is due to the proper effect of the sulfonate groups and the impact of the bi-functionality. The two REEs have comparable SC values, and neither of the two sorbents can be used to directly separate the two metals. The improvement in the selective separation by sulfonation (especially at the highest pH values) is less marked in the case of Fe(III). The selectivity coefficients for MC-UR are less influenced by the pH than those for MC-UR/S. Based on the SC_Ce/metal_ and SC_Nd/metal_, the preferential sorption follows the trends below:

For MC-UR: Ce(III) > Nd(III) > Fe(III) > Al(III) > Zn(II) > Mg(II) > Ca(II)

For MC-UR/S: Ce(III) > Nd(III) > Fe(III) > Zn(II) > Mg(II) > Al(III) > Ca(II).

The sorption capacities are plotted vs. the Shannon ionic radius in Figure S16 at different pH values for both the MC-UR and MC-UR/S sorbents. Irregular (parabolic) trends appear in the acidic solutions. At pH 4.21–5.19 for MC-UR and at pH 3.89–4.75 for MC-UR/S, the sorption capacities linearly increase with the radius of the trivalent metal cations, while the opposite trend is observed for the divalent cations. Surprisingly, in the case of MC-UR/S at pH 3.21, all the sorption capacities follow a linear increasing trend with the Shannon ionic radius (without the breaking difference between the trivalent and divalent cations).

Figure S17 explores another type of correlation plotting for the divalent and trivalent cations, the sorption capacities vs. the ionic index (i.e., Z^2^/r, where Z is the ionic charge of the metal ion and r is its ionic radius). The data were collected from the two sets of pH series, identified in Figure S12 (as linear trends). The two groups of metal ions show relatively good correlations with both MC-UR and MC-UR/S. The same test was performed by plotting the sorption capacities vs. the covalent index (i.e., X_m_^2^ * r, where X_m_ is the Pauling electronegativity, not shown). Herein, it was not possible to identify a clear correlation between the parameters. Apparently, the binding of the metal ions mainly involves ionic interactions rather than covalent bonds.

It is noteworthy that cerium sulfate was used for the study of sorption using multi-component solutions. The control of the pH with HCl and the co-existence of competitor metals, as chloride salts, contribute to maintain at least a fraction of the cerium as cerium chloride cations (i.e., CeCl^2+^) in complement to Ce(SO_4_)_2_^−^. The residual concentration of Ce(III) is close to 0.4 mmol Ce L^−1^, with a sorption capacity close to 0.67 mmol Ce g^−1^. Compared with the sorption capacity at C_eq_: 0.4 mmol Ce L^−1^ in the sorption isotherm (i.e., ≈1.5 mmol Ce g^−1^), the sorption was strongly reduced (by more than 2 times). This decrease may thus be due to both the competition effects of other metals bound simultaneously and the impact of changes in the metal speciation (due to the cerium sulfate salt).

#### 3.2.5. Metal Desorption and Sorbent Recycling

As the sorption of cerium is favored at pH 5, acidic solutions are logically good candidates for reversing the binding of the metal from loaded sorbents. This is confirmed by a scan of the literature on cerium sorption, as 0.2 M HCl solutions were successfully used for Ce(III) desorption from metal-bound biosorbents [26,79,80]. Vijayaraghavan et al. [28] used 0.05 M HCl solutions in the case of brown algal biomass. Hamza et al. [48] used a combination of HCl and CaCl_2_ (0.5 M/0.2 M CaCl_2_ solution) for the elution of REEs from algal/PEI beads (calcium chloride contributes to the stabilizing of the alginate network). Kołodyńska et al. [31] used zero-valent iron biochar composites for the sorption of REEs, and they found that molar acidic solutions successfully desorb rare earths with a preference for Ce(III) > Nd(III) > La(III), with HNO_3_ being more efficient (82–95%) than HCl (80–93%) and H_2_SO_4_ (77–86%). Hamza et al. [81], using another type of magnetic chitosan derivative, showed that even with a 0.5 HCl solution, the release of iron does not exceed 1.1%, meaning that the magnetite remains relatively stable. Herein, the HCl concentration was set to 0.2 M to limit the possible leakage of the iron and degradation of the magnetite. Figure S18 compares the Ce(III) desorption kinetics of MC, MC-UR, and MC-UR/S. The metal is completely desorbed within 20–40 min, and the required time increases according to the order of: MC-UR/S < MC < MC-UR. This is slightly faster than the proper uptake kinetics.

After demonstrating the effectiveness of the elution, the recycling of the sorbent was evaluated over five successive cycles of sorption and desorption (Table 1). Comparing the sorption and desorption efficiencies, we can see that the cerium is systematically, completely eluted, meaning that the metal is not accumulated during the sorption lifecycle. On the other hand, the sorption efficiency progressively decreases. However, the loss in the metal recovery remains lower than 9% over the fifth cycle for the MC sorbent. The stability is even better in the case of the functionalized sorbents; the loss in cerium removal is diminished by 3.9% and 3.1% for MC-UR and MC-UR/S, respectively. The functionalization enhances the stability of the sorption performance.

The weak loss in the sorption efficiency is apparently not associated with the progressive saturation of the sorbent, since desorption is complete. Therefore, this may be due to physicochemical changes derived from the sorbent processing. The successive incidences of contact with pH 5 and the 0.2 M HCl solutions may cause some alterations in the sorbents. The FTIR spectra of the sorbents after metal desorption (in the fifth cycle) were compared with the spectra of the original materials but also with their spectra after being exposed to the 0.2 M HCl solution (to isolate the respective contributions of the desorption and pH change, see Section SB in the Supplementary Materials and Figure 1).

#### 3.2.6. Application to Ore Raffinate

The ore was collected in the Abu Zenima area (in Southwestern Sinai, Egypt, Figure S19) in a mineralogical Adedia formation (described in Section SD). This ore is mainly comprised of phosphate minerals (iron, aluminum, magnesium, and rare earths) associated with aluminosilicate (Table S9). In addition to these major elements, the mineralization is also characterized by noticeable amounts of titanium, uranium, and lead.

The acidic leaching of the ore was processed at 150 °C in an agitated tank reactor for 2 h using concentrated sulfuric acid (200 g H_2_SO_4_ L^−1^). The solid/liquid ratio was set to 1/3. The composition of the leachate is summarized in Table S10. The most remarkable concentrations were those of iron (i.e., 20.8 g Fe L^−1^), aluminum (i.e., 8.17 g Al L^−1^), calcium (i.e., 1.17 g Ca L^−1^), and manganese (i.e., 0.98 g Mn L^−1^). The acidic leachate also contains high concentrations of REEs, with the global REE index being close to 8.7 g REE L^−1^, which is, notably, comprised of neodymium (i.e., 0.98 g Nd L^−1^) and cerium (i.e., 0.43 g Ce L^−1^).

Uranium is another important element identified in the leachate (i.e., 200 mg U L^−1^), which serves to decrease the possible contamination of the valorized REEs with hazardous radio-elements (potentially including thorium). A pre-treatment of the leachate was conducted at pH 1.8 using an Amberlite IRA-400 column (quaternary ammonium salt resin). The uranyl removal reached 81%, while the loss in the REEs and cerium was less than 5% (up to 11% for neodymium).

The high concentration of iron in the residue of the sorption may interfere with the further steps in the valorization of strategic metals. After controlling the pH to 4, 98.8% of the residual iron was precipitated (global loss, GL: 98.9%). This step hardly changed the residual concentrations of U (GL: 83.5%) and the REEs (GL: 9.3%), including 10.1% for Ce(III) and 18.1% for Nd(III).

The next step in the process consists of the recovery of the REEs using a strong cation exchange resin (bearing sulfonic groups), Dowex 50X8, at pH 4. The resin collected 93.7% of the REEs contained in the residue of the pH 4 precipitation step, and 84.8% for Ce(III) and 87.2% for Nd(III) (Table S11). The residual concentrations of REEs stand at 498.6 mg REE L^−1^ (58.8 mg Ce L^−1^ and 102.7 mg Nd L^−1^). 

About 38.8% of the initial aluminum content remained in the outlet of the Dowex 50X8 column, and the residual concentration reaches about 5 g Al L^−1^. A new precipitation step was processed at pH 5 to remove the aluminum (to limit its competition effect in the next sorption step while using MC-UR/S). After this step, the uranium concentration was barely changed, while the residual concentrations of the REEs remained close to 211 mg REE L^−1^, 57 mg Nd L^−1^ and 32.1 mg Ce L^−1^).

Figure S20 summarizes the distribution of the main relevant elements in the different compartments of the pre-treatment leachates. Rare earth elements are mainly accumulated in the Dowex 50X8 sulfonic resin (71.5–85.0%), which also binds lead (21.2%), while the relative fractions of the other metals are systematically lower than 7.2%. Iron is mainly present in the precipitate collected at pH 4 (i.e., 94.5%). The precipitation at pH 5 recovers 59.3% of Al(III), 39.4% of Cu(II), 17.7% of Mn(II), and 12.0% of Ca(II). The quaternary ammonium salt resin Amberlite IRA-400 recovers 81.1% of U(VI) (14.8% remains in the raffinate, to be treated with MC-UR/S), and up to 20.6% of Si(IV) is bound to the resin, while for the other metal ions, the relative fractions remain below 10.6%. In fact, the raffinate contains appreciable fractions of Ni(II) (80.4%), Si(IV) (i.e., 66.7%), Pb(II) (i.e., 53.8%), Ca(II) (i.e., 50.9%), and Cu(II) (i.e., 40.0%), and 14.8% U(VI) (referring to the amounts present in the leachate).

The raffinate contains limited values of REEs (between 2.4 and 7.5%), and the residual concentrations are limited to 32.1 mg Ce L^−1^ (i.e., 0.242 mmol Ce L^−1^) and 57.4 mg Nd L^−1^ (i.e., 0.398 mmol Nd L^−1^). These concentration ranges are compatible with the conditions for the effective application of MC-UR/S. The treatment of the raffinate solution was performed at different pH values. Figure S21 shows that for all the elements, the sorption capacity increases with the pH of the solution. Under the most favorable conditions (i.e., pH_eq_: 4.87), the recovery of metal ions from the raffinate reaches 59.4% for Ce(III) and 38.7% for Nd(III) (60.4% for the global REE index). Uranyl was also co-extracted (SE: 33.6%) together with Fe(III) (SE: 32.7%).

Figure S22 reports the log_10_ plot of the distribution ratio vs. pH_eq_. The rare earth elements show higher relative distribution ratios than the other metal ions or metalloids. The sensitivity to pH is demonstrated by the slopes of the plots: the highest sensitivities to pH are reported for Ce(III) > U(VI) > Pb(II) > Fe(III) > Nd(III) > Ca(II) ≈ Ni(II) > Al(III) ≈ Si(IV) > Mn(II). There is no direct correlation between this ranking and the intrinsic physicochemical properties of the target metals. The effects of the differential concentration levels cannot apply to the relationships established above for the equimolar multi-component solutions (in Section 3.2.4).

The comparison of the molar distribution of the main elements in the raffinate (Figure S23a) and in the residue of the sorption using MC-UR/S at the optimum pH (Figure S23b) shows that, for most of the target elements, the molar fraction increases with the sorption. The extent depends on the metal or metalloid, according to the following series:

Pb(II) > Ce(III) ≈ Fe(III) > U(VI) >> Mn(II) > Al(III) ≈ Ni(II) >> Nd(III) >> Ca(II) ≈ Si(IV).

This scale can be used to evaluate the potential of the sorbent to selectively enrich some metals on the sorbent.

Figure S24 compares the plots of the selectivity coefficients SC_Ce/metal_ and SC_Nd/metal_ at different pH values. The sorbent has a marked preference for Ce(III) against the heavy metals. This preference is more marked than that for Nd(III). The SC values are globally better at pH_eq_ 4.15, which represents a good compromise, taking into account both the sorption capacities and efficiencies, as well as the selectivity parameters. The exception is the selectivity of MC-UR/S against Pb(II), which is maximal at pH 2.19, considering both Ce(III) and Nd(III).

This work focused on the recovery of cerium from processed leachate using MC-UR/S. It would be easy to recover the REE through acidic desorption (as illustrated by Section 3.2.5) and then process the eluate by specific precipitation. Indeed, the metal could readily be recovered using oxalic acid for the precipitation of the REE under controlled pH conditions (i.e., pH 1.5) [82,83].

Han and Kim [84] reported that at a low H_2_SO_4_ concentration (0.1–0.2 M), CeSO_4_^+^ and Ce(SO_4_)_2_^−^ may coexist, while at a higher concentration (>1 M), the only species present in the solution is the anionic form. Obviously, the environmental conditions may affect the affinity of the sorbent for cerium through variations in the metal speciation. Under the conditions selected for the tests on the processed ore leachate, cerium may be present at least partially under a different speciation than that in the preceding study (where the metal exists as CeCl^2+^). Therefore, the decrease in the sorption performance, compared with the synthetic solutions, may be due to the cross-effects of the competition with other metals for binding on the reactive groups and the change in the speciation (charge of the cerium species).

## 4. Materials and Methods

### 4.1. Materials

Sodium bisulfite ~40%, formaldehyde 38% in H_2_O, chitosan (25% degree of acetylation, DA), epichlorohydrin (EPI) 98%, urea >99%, NaOH anhydrous ≥98%, calcium chloride anhydrous >97%, glutaraldehyde solution (25% *w/w*), and sodium nitrite ≥99.0% were supplied by Sigma-Aldrich (Merck-KGa, Darmstadt, Germany). Neodymium sulfate (used in the selectivity experiments) and cerium sulfate were provided by the National Engineering Research Center of Rare Earth Metallurgy and Functional Materials Co., Ltd. (Baotou, China). MgCl_2_·6H_2_O, AlCl_3_·6H_2_O, CuSO_4_, and ZnCl_2_ were obtained from Guangdong Guanghua, Sci-Tech Co., Ltd. (Guangdong, China).

### 4.2. Synthesis of Sorbents

**Scheme 2:**
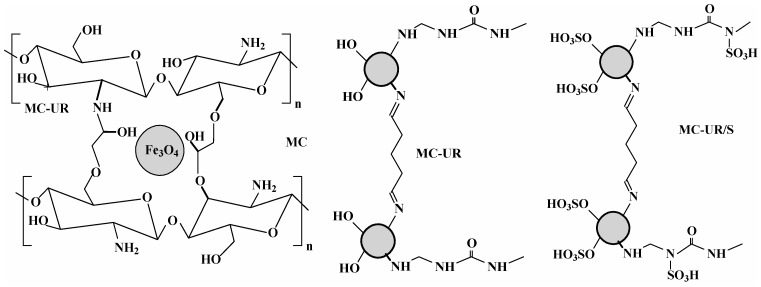
Suggested structures of the sorbents.

The synthesis of the sorbents includes a series of steps that are described in detail in the Supplementary Information (Section SE). Briefly, the magnetite nanoparticles were prepared by the thermal co-precipitation of Fe(II) and Fe(III) salts. The magnetite chitosan particles were obtained by the dispersion of the magnetite nanoparticles into a chitosan solution. After adding glutaraldehyde to the suspension, the magnetic chitosan (MC) microparticles were collected. For the synthesis of MC-UR, we mixed the magnetite nanoparticles with urea and formaldehyde before mixing them with a chitosan acid solution and adding, in the last step, glutaraldehyde (for the crosslinking). A sulfonating agent (N(SO_3_Na)_3_) was prepared through a controlled reaction of sodium bisulfite with sodium nitrite in solution. This reagent was used for sulfonating the MC-UR microparticles and producing (at a controlled temperature) MC-UR/S. Scheme 2 reports the suggested structures of the selected sorbents.

### 4.3. Characterization of Sorbents

The morphology of the sorbents was observed by SEM analysis (Phenom-ProX SEM Eindhoven-Netherlands-Thermo Fisher Scientific, Waltham, MA, USA), while the chemical composition was obtained by semi-quantitative EDX analysis (coupled with SEM). FT-IR spectra were collected for the sorbents at different stages of use (raw, after metal sorption, and after the fifth desorption). The samples were incorporated into KBr pellets and analyzed using an IR-Tracer 100 FT-IR (Shimadzu, Tokyo, Japan). The thermogravimetric properties were characterized using a Netzsch STA-449-F3 Jupiter thermal analyzer (Netzsch-Gerätebau-HGmbh; Selb, Germany) under nitrogen, with a ramp of 10 °C min^−1^. The BET-surface area and porous characteristics were collected through the acquisition of nitrogen adsorption–desorption isotherms (Micromeritics-TriStar, II-Norcross, USA-system, 77 K) using the BET and BJH equations. The samples were swept at 120 °C under N_2_ for 4 h. The size of the sorbent particles was determined by TEM analysis (JEOL, 1010- JEOL Ltd., Tokyo, Japan). The pH_pzc_ (pH of zero charge) was performed by the pH drift method [85]. The pH was measured using a compact pH ionometer, S220 Seven (Mettler-Toledo, Shanghai, China). Filtration was performed on the samples before the analysis using 1.2 µm membranes. The elements were measured using ICP-AES (ICPS-7510, Shimadzu, Tokyo-Japan). The magnetic properties (M-H loop) were analyzed using a Lake Shore 7410 vibrating sample magnetometer (VSM, Lake Shore Cryotronics, Westervill, OH, USA). The elemental composition (C, S, N, H, and O weight contents) analysis was carried out on the sorbent using an element analyzer (CHNOS, Vario EL III, Elementar Analysensysteme GmbH, Sonaustraβe, Langenselbold, Germany).

### 4.4. Sorption Studies

Sorption studies were systematically performed in batch systems. A fixed volume of the solution (V, L) containing a given concentration of metal (C_0_, mmol L^−1^) was mixed with a fixed amount of the sorbent (m, g). The experiments were carried out at room temperature (i.e., 20 ± 1 °C) under agitation (at 210 rpm). The initial pH was controlled by sulfuric acid and NaOH solutions (1/0.1 M). Although the pH was not controlled during sorption, the equilibrium pH (i.e., pH_eq_) was systematically monitored at the end of the experiments. At given contact times (for uptake kinetics) or at equilibrium (24–48 h), the samples were collected by filtration (and/or magnetic separation), and the residual metal concentration (C(t) or C_eq_, mmol L^−1^) was analyzed by inductively coupled plasma atomic emission spectrometry (ICPS-7510, Shimadzu, Tokyo, Japan). The mass balance equation enables the calculation of the sorption capacity (q, mmol g^−1^): q = (C_0_ − C_eq_) × V/m. The same protocol was used for the experiments involving the presence of different metals or salt (i.e., Na, Cl, etc.). For the study of the cerium desorption, the metal-loaded sorbents (collected from the uptake kinetics experiments) were also processed in batch systems in the presence of 0.2 M HCl solutions. Water-rinsing steps were systematically applied between the sorption and desorption operations. The sorption tests were triplicated. The extensive experimental conditions are systematically reported in the captions of the figures.

In the final application, the cerium recovery was investigated using MC-UR/S in pre-treated solutions resulting from the leaching of an Egyptian ore (sulfuric acid concentrated solutions). A series of pre-treatments were processed on the acidic leachate, including U recovery using the Amberlite IRA-400 ion exchange resin (in fixed-bed column), pH control (at pH 4 for the partial precipitation and co-precipitation of the base metals), and REE recovery using the Dowex 50 X8 ion exchange resin. The outlet of the resin column was controlled at pH 5 (to precipitate mainly Al(III)) before carrying out the sorption tests on this raffinate using MC-UR/S (at different pH values) in an agitated reactor. The detailed presentation of the experimental procedures is reported in the Supplementary Materials.

### 4.5. Modeling of the Sorption Properties

Conventional equations were used to model the uptake kinetics (including the pseudo-first- and pseudo-second-order rate equations, and the Crank equation, Table S12a) and sorption isotherms (including Langmuir, Freundlich, Sips, Temkin and Dubinin–Raduskevich equations, Table S12b). The determination coefficient and the Akaike information criterion (AIC) were systematically determined to compare the fitting of the experimental data with these models. Non-linear regression analysis (Mathematica^®^, Wolfram Research, Champaign, IL, USA) was systematically applied to optimize the selection of the fitting parameters.

## 5. Conclusions

This work demonstrates that simple procedures for the functionalization of magnetic chitosan microparticles (≈6 µm in size) can be used to successively immobilize amine groups and grafted sulfonate moieties (confirmed by elemental analysis: ≈4.6 mmol N g^−1^, and 1 mmol S g^−1^, when relevant).

The FTIR analysis confirms the contributions of different reactive groups to the binding of the metal (amine/amide and hydroxyl/carbonyl from the chitosan and urea, and S-based groups after the sulfonation of the sorbent). The magnetite nanoparticles (5–7 nm) embedded in the polymer matrix confers superparamagnetic properties on the composite (MC-UR/S).

The sorption capacity at pH_0_ 5 doubled for the aminated sorbent and tripled after dual functionalization (amination and sulfonation). The improvement in the sorption performance is not correlated with the density of the reactive groups, and this enhancement also results from the synergetic effects of the bi-functionalization. The fast kinetics (though partially controlled by the resistance to intraparticle diffusion) enable the reaching of the equilibrium within 60 min of contact, and the kinetic profiles are effectively described by the pseudo-first-order rate equation. The acidic solutions (0.2 M HCl) are highly efficient in the cerium elution from the metal-loaded sorbents. Despite the changes observed in the FTIR spectra after the metal desorption and sorbent recycling (in the fifth cycle), the comparisons of the desorption efficiency (which remained close to 100%) and sorption efficiency (which lost only 3–4% for the functionalized sorbent vs. 11% for the raw MC) confirm the outstanding stability of the functionalized sorbents.

The study of the pH effect on the metal sorption using the equimolar multi-component solutions shows that the functionalized sorbents have a marked preference for rare earth elements against heavy metals in mild acidic solutions. The sorption performances are correlated with the intrinsic physicochemical characteristics of the metals; the sorption capacities for the grouped metals (according to their ionic charge) are correlated with their ionic index as an indication of the preference for ionic interaction rather than covalent bonding.

The acidic leachate of an Egyptian ore generates leachates containing high concentrations of Al, Fe, and U, REEs that can be pre-treated using resins for the recovery of U (Amberlite IRA-400) and REEs (Dowex 50X 8) and for the removal of excessive amounts of Al and Fe (by precipitation). However, the effluent still contains appreciable quantities of REEs. In order to valorize this effluent, MC-UR/S was successfully applied for the sustainable recovery of REEs. We observed that the sorbent has a great selectivity against heavy metals, especially at a pH_eq_ of around 4.2 (iron being the least efficiently separated within this series of competitor ions).

## Figures and Tables

**Table 1:** Reuse of sorbents in the recovery of Ce(III)—sorption (SE, %) and desorption (DE, %) efficiencies for five successive cycles.

**Cycle**		**MC**		**MC-UR**		**MC-UR/S**	
		**SE (%)**	**DE (%)**	**SE (%)**	**DE (%)**	**SE (%)**	**DE (%)**
1	Aver.	16.1	100.6	45.9	99.9	71.8	100.4
	Std. Dev.	0.6	0.5	0.5	1.1	1.5	0.5
2	Aver.	15.6	99.8	45.2	100.3	71.7	100.2
	Std. Dev.	0.4	0.7	0.4	0.1	1.5	0.9
3	Aver.	15.5	100.3	44.9	100.6	70.8	99.7
	Std. Dev.	0.6	0.2	0.7	0.4	1.1	0.5
4	Aver.	15.1	100.5	44.6	100.0	70.2	100.0
	Std. Dev.	0.7	0.5	1.0	0.9	0.8	0.5
5	Aver.	14.7	99.9	44.2	100.0	69.7	100.3
	Std. Dev.	0.7	0.4	0.8	0.5	0.8	0.2
Loss (5th/1st) (%)		8.7	-	3.8	-	3.1	-

Exp. Cond. for sorption—C_0_: 0.366 mmol Ce L^−1^; pH_0_: 5; SD: 0.4 g L^−1^; time: 24 h; v: 210 rpm; T: 22 ± 1 °C. Exp. Cond. for desorption—eluent: 0.2 M HCl; SD: 2 g L^−1^; time: 2 h; v: 210 rpm; T: 22 ± 1 °C.

## Data Availability

Data can be obtained from the authors.

## Supplementary Materials

The following supporting information can be downloaded at: https://www.mdpi.com/article/10.3390/molecules27217562/s1, References [23,27,29,33,34,47,48,51,52,68,74,75,76,77,78,79,86,87,88,89,90,91,92,93,94,95,96,97,98,99,100,101,102,103,104,105,106,107,108,109,110,111,112,113,114,115,116,117,118,119,120,121,122,123,124,125,126,127,128,129,130,131,132,133,134,135,136,137,138] are cited in the supplementary materials. Table S1. Examples of functionalized sorbents for rare earth recovery. Table S2a. FTIR spectra of the sorbents (MC-UR, MC-UR/S) before (raw) and after Ce(III) sorption (+Ce(III)) and after the fifth desorption (5th Des.). Table S2b. FTIR spectra of the sorbents (MC-UR and MC-UR/S) before (raw) and after Ce(III) sorption (+Ce(III)) and after conditioning at pH 5. Table S2c. FTIR spectra of the sorbents (MC-UR and MC-UR/S) before (raw) and after five cycles of sorption and desorption, and after conditioning with 0.2 M HCl. Table S3. Elemental analysis of the sorbents. Table S4. Uptake kinetics for Ce(III) sorption using the MC, MC-UR, and MC-UR/S sorbents—parameters of the models. Table S5. Ce(III) sorption isotherm using MC—parameters of the models. Table S6. Ce(III) sorption isotherm using MC-UR—parameters of the models. Table S7. Ce(III) sorption isotherm using MC-UR/S—parameters of the models. Table S8. Comparison of Ce(III) sorption properties with alternative sorbents. Table S9. Composition of the ore. Table S10. Pre-treatment of ore leachate—concentrations (mg L−1) of the selected metals. Table S11. Composition of ore leachate, raffinate (including metal recovery efficiency, RE (%)) and residue of adsorption onto MC-UR/S at different pHeq values. Table S12a. Reminder of the equations used for modeling the uptake kinetics. Table S12b. Reminder of the equations used for modeling the sorption isotherms. Figure S1. SEM image for the MC-UR/S sorbent. Figure S2. TEM images of the MC (a), MC-UR (b), and MC-UR/S (c) sorbents. Figure S3. VSM analysis of the MC-UR/S sorbent. Figure S4. TGA analysis of the MC (a), MC-UR (b), and MC-UR/S (c) sorbents. Figure S5. DrTG analysis of the MC (a), MC-UR (b), and MC-UR/S (c) sorbents. Figure S6. Effect of pH 5 on the FTIR spectrum of MC-UR (compared with the reference sorbent and Ce-loaded MC-UR). Figure S7. Effect of pH 5 on the FTIR spectrum of MC-UR/S (compared with the reference sorbent and Ce-loaded MC-UR/S). Figure S8. Effect of acidic conditions (0.2 M HCl solution) on the FTIR spectrum of MC-UR (compared with the reference sorbent and MC-UR after the fifth sorption desorption). Figure S9. Effect of acidic conditions (0.2 M HCl solution) on the FTIR spectrum of MC-UR (compared with the reference sorbent and MC-UR after the fifth sorption desorption). Figure S10. Determination of pHPZC for the MC, MC-UR, and MC-UR/S sorbents. Figure S11. Ce(III) speciation diagram. Figure S12. pH variation during Ce(III) sorption using the MC, MC-UR, and MC-UR/S sorbents. Figure S13. Distribution ratio as a function of pHeq (log10 plot) for Ce(III) sorption using the MC, MC-UR, and MC-UR/S sorbents. Figure S14. Effect of pHeq on the distribution ratio (log10 unit) using the MC-UR (a) and MC-UR/S (b) sorbents from the sorption tests on the multicomponent equimolar solutions. Figure S15. Selectivity tests for Nd(III) sorption using the MC-UR (a) and MC-UR/S (b) sorbents with the multicomponent equimolar solutions—effect of pHeq on SCNd/metal. Figure S16. Correlation of the sorption with the Shannon ionic radius for the metal sorption in multicomponent solutions at different pH values. Figure S17. Correlations between the sorption capacities and ionic index for the sorption of metal ions from the multi-component equimolar solutions using MC-UR (a) and MC-UR/S (b). Figure S18. Ce(III) desorption for the metal-loaded MC, MC-UR, and MC-UR/S sorbents. Figure S19. Geological map of the studied area. Figure S20. Distribution of main elements in the different compartments of the pre-treatment of leachates. Figure S21. Effect of pH on the metal recovery and sorption capacity for the selected elements from ore raffinate using MC-UR/S—sorption capacities (qeq), maximum sorption efficiency (R(%) at optimum pHeq, i.e., 4.87), and reference initial metal concentration in the raffinate. Figure S22. Metal recovery from ore raffinate using the MC-UR/S sorbent—effect of pHeq on the distribution ratio (log10 plot). Figure S23. Molar distribution of main elements in the raffinate (a) and the residue of the sorption step at pHeq 4.87 (b). Figure S24. Effect of pH on the selectivity coefficients for the metal sorption from ore raffinate—(a) SCCe_/metal_ and (b) SC_Nd/metal_.
