# Supplementary material for: Enhancement of Cerium Sorption onto Urea-Functionalized Magnetite Chitosan Microparticles by Sorbent Sulfonation—Application to Ore Leachate
Source: Molecules. 2022 Nov 4;27(21):7562. doi: 10.3390/molecules27217562 (PMC9653577; doi:10.3390/molecules27217562)
Supplement: Supplementary file 1 [file molecules-27-07562-s001.zip › molecules-2005434-supplementary.pdf]

Enhancement of cerium sorption onto urea-functionalized magnetite chitosan microparticles by sorbent sulfonation – Application to ore leachate

Mohammed F. Hamza [1,2], Eric Guibal [3*], Adel A.-H. Abdel-Rahman [4], Marwa Salem[4],

Mahmoud S. Khalafalla,[2] Yuezhou Wei [1,5], and Xiangbiao Yin [1*]

1 School of Nuclear Science and Technology, University of South China, Heng Yang 421001, China
2 Nuclear Materials Authority, POB 530, El-Maadi, Cairo, Egypt
3 Polymers Composites and Hybrids (PCH), IMT Mines Ales, Alès, France
4 Chemistry Department, Faculty of Science, Menofia University, Shebin El-Kom, 32511 Egypt
5 School of Nuclear Science and Engineering, Shanghai Jiao Tong University, Shanghai, China
* Correspondence: eric.guibal@mines-ales.fr (EG, +33(0)466782734), yinxb@usc.edu.cn (XY)

Supplementary Information

Section SA. Rationales for the synthesis of sorbents in relation with alternative REEs sorbents

Table S1. Examples of functionalized sorbents for rare earth recovery.

Sorbent	Functional groups	Metal	pH	$q_{m,L}$	Ref.
Amidoximated algal/PEI beads	Amine, Amidoxime	La	6	0.547	[1]
		Dy	6	0.576	
Phosphorylated Algal/PEI	Amine, Phosphoryl	Nd(III)	5	1.46	[2]
		Mo(VI)	3	2.09	
Sulfonated Algal/PEI	Sulfonic	Sc(III)	4	3.16	[3]
		Ce(III)	4	0.71	
		Ho(III)	4	0.61	
GO/poly(isopropylacrylamide-maleic acid) cryogel	Amine, Carboxylic	La(III)	5	0.237	[4]
Funct. magnetic β-cyclodextrin polymer	Tetrafluoroterephthalonitrile	Nd(III)	5	0.062	[5]
		Gd(III)	5	0.049	
N-doped carbon dots/alginate	Amine, Carboxylic, Hydroxyl	Dy(III)	5	1.28	[6]
		Sm(III)	5	1.23	
		Pr(III)	5	1.23	
Polystyrene-poly(hydroxamic acid)	Hydroxamic	La(III)	2	1.27	[7]
		Ce(III)	1	1.53	
		Y(III)	3	1.83	
PAN/SiO_2	Pyridylazo-naphtol	Tb(III)	8	0.732	[8]
Imprinted carboxymethyl chitosan	Amine, Carboxylic	Gd(III)	7	0.161	[9]
Aminated polymeric sorbent	Amine	Nd(III) Gd(III) Ho(III)	6.5	-	[10]
DTPADA-aminated polymer	Diethylenepentaacetic		2.5	0.018	
PAA-aminated polymer	Phosphonoacetic		6.5	0.032	
BPG-aminated polymer	Phosphonomethylglycine		6.5	0.019	
Functionalized corn stalk gels	Carboxylic	Nd(III)	3	2.44	[11]
Benzene triamido-tetraphosphonic SiO_2	Amido, Phosphonic	Nd(III)	6	0.90	[12]
		La(III)	6	0.191	
		Ce(III)	6	0.244	
		Y(III)	6	0.987	
Poly-γ-glutamate-Na	Amine, Carboxylic	Nd(III)	3	1.57	[13]
Poly(hydroxamic acid) cellulose/PMA	Hydroxamic	La(III)	6	1.86	[14]
		Ce(III)	6	1.74	
		Pr(III)	6	1.67	
		Gd(III)	6	1.39	
		Nd(III)	6	1.44	
EDTA β-cyclodextrin	Amine, Carboxylic	La(III)	4	0.343	[15]
		Ce(III)	4	0.353	
		Eu(III)	4	0.365	
Hydrolyzed poly(styrene-co-maleic anhydride)	Di-carboxylic	La(III)	6	2.06	[16]
		Eu(III)	6	1.99	
		Tb(III)	6	1.92	
		Yb(III)	6	1.95	
Alginate	Carboxylic	La(III)	5	0.590	[17]
		Ce(III)	5	0.649	
Poly-γ-glutamate-Na/alginate	Amine, Carboxylic	La(III)	5	1.18	
		Ce(III)	5	1.10	

Magnetic DETA-chitosan	Amine	Nd(III)	5	0.352	[18]
		Dy(III)	5	0.315	
		Yb(III)	5	0.303	
Magnetic cysteine-chitosan	Carboxylic, Amine, Sulfur	La(III)	5	0.115	[19]
		Nd(III)	5	0.106	
		Yb(III)	5	0.103	

Units: q_m, mmol g^{-1}.

Section SB. Characterization of sorbents

Figure S1 shows the scanning electron microscopy (SEM) image of sorbent particles, which are characterized by irregular shapes and length. By image analysis, it was possible evaluating the size of MC-UR/S close to 6 ±4 µm. The objects are elongated with rounded edges. The transmission electron microscopy (TEM) analysis (Figure S2) shows the embedment of dense magnetic nanoparticles into rounded irregular polymeric objects; the size of the composite nano-objects is around 5-7 nm. The encapsulation of magnetite nanoparticles with chitosan prevents their aggregation. However, at the end of the synthesis (including drying step), MC-UR/S micro-particles appear as dense objects (as shown on SEM picture).

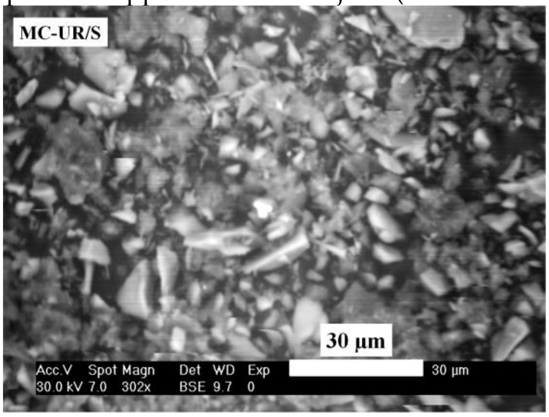

Figure S1. SEM image for MC-UR/S sorbent.

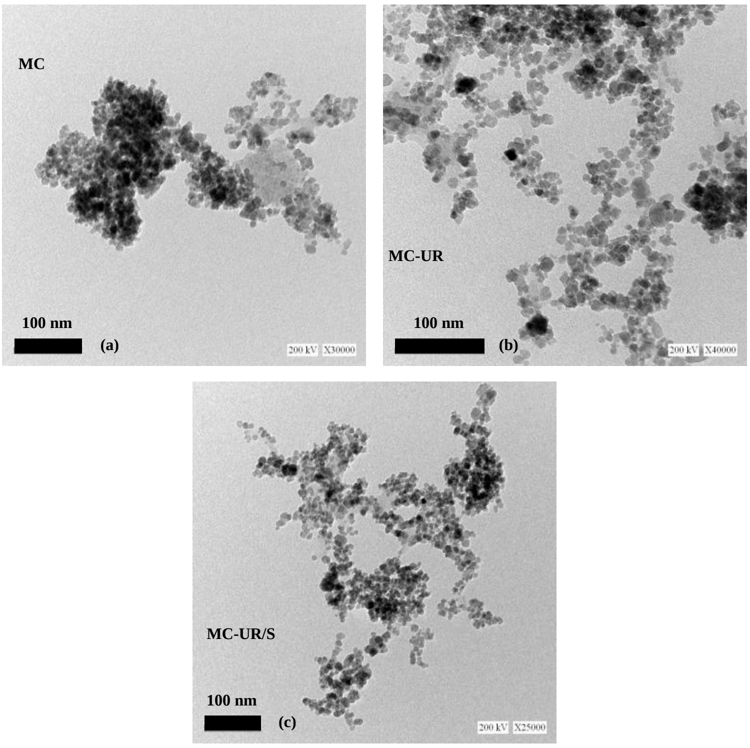

Figure S2. TEM images of MC (a), MC-UR (b), and MC-UR/S (c) sorbents.

In Figure S3, the vibrating sample magnetometry (VSM) reports the hysteresis cycle for the magnetization of MC-UR/S sorbent. The saturation magnetization (M_s) reaches about 18.7 emu g^{-1}; this value is high enough to making possible the separation of sorbent particles from liquid phase. The saturation magnetization is much lower than the value usually reported for magnetite nanoparticles (i.e., ≈93 emu g^{-1}) [20]. The coating of magnetite NPs with polymer may exert a double effect through (a) the shielding of magnetite that reduces the magnetization, and (b) the reduction of the magnetite proportion in the composite (herein chitosan being non-magnetic gives a “dilution” effect when associated with magnetite). This value is of the same order of magnitude than the Ms of magnetic-chitosan composite developed for dye binding by Ibanescu et al. [21]. Bezdorozhev et al. [22] compiled the magnetic properties of a series of magnetic-chitosan

composites, and they highlighted the strong influence of processing conditions on the morphology of particles and on M_s values. Lei et al. [23] reported the progressive Ms decrease when magnetite nanoparticles are incorporated in to chitosan and further chemically modified (by carboxymethylation of the biopolymer: ≈55 > ≈48 > ≈14, respectively). Cheraghipour et al. [24] succeeded in maintaining high levels of magnetization (around 62 emu g^{-1}) for magnetite (stabilized by citric acid) after being conjugated with chitosan (76 emu g^{-1} before chitosan-immobilization). It is noteworthy that MC-UR/S may be classified as superparamagnetic; indeed, the hysteresis loop is very weak: the coercivity and the remanence are hardly detectable; in addition, the slope of Moment/Mass *vs.* Field is very steep.

Herein, the loss in magnetization is mainly associated to the weak amount of magnetite into the composite material. This is confirmed by the TGA analysis of the sorbents (at the different stages in the production). The total weight loss mainly corresponds (at T: 800 °C) to the residual fraction of mineral (magnetite) into the composite: 44.0%, 54.8% and 73.6% for MC, MC-UR and MC-UR/S, respectively. Therefore, in MC-UR/S the magnetite content can be roughly evaluated to 26%; this would make the Ms consistent with the re-calculated value (i.e., ≈23 *vs.* ≈19 emu g^{-1}).

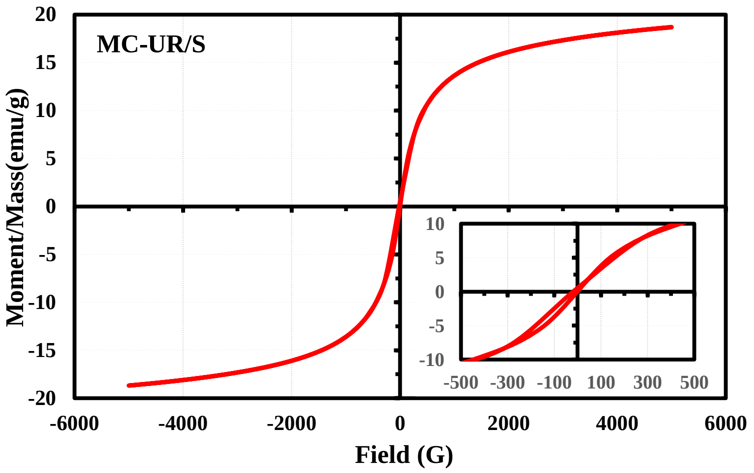

Figure S3. VSM analysis of MC-UR/S sorbent.

The TGA analysis of the three sorbents (MC, MC-UR, and MC-UR/S) shows roughly the same trends described by three main degradation waves:

1. Release of water sorbed at the surface of the sorbent (weight loss, WL: ≈10-12.5%), in the range 30 to 224-248 °C,
2. Depolymerization of chitosan, degradation of amine groups (including from thiourea in the case of substituted biopolymer) (WL: ≈22-30%), in the range 224-248 °C to 420-509 °C. It is noteworthy that the onset temperature for this degradation steps decreases with the level of substitution of chitosan (i.e., crosslinked chitosan in MC > MC-UR > MC-UR/S),
3. Char formation and degradation appears above 420-509 °C (depending on the sorbent) with WL close varying between 10.0-12.7% (for MC and MC-UR) and up to 39.9% for MC-UR/S.

The residue (mainly constituted of magnetite) represents 56.0%, 45.2%, and 26.4% for MC, MC-UR, and MC-UR/S, respectively. In some cases, additional small and poorly-resolved intermediary waves also appear; however, they are better identified after DrTG. In the case of MC, three well-marked peaks are identified at 64.5 °C, 247.1 °C, and 546 °C (plus a shoulder at 295.4 °C). Two main peaks are observed at 71.0 °C and 286.7 °C (completed by two weak peaks at 534.6 °C and 574.6 °C) on MC-UR DrTG curve. Three peaks (with increasing intensity) at 74.7 °C, ≈280 °C (broad), and 471.5 °C appear in the profile of MC-UR/S sorbent. The stability decreases with the substitution of (crosslinked) MC composite.

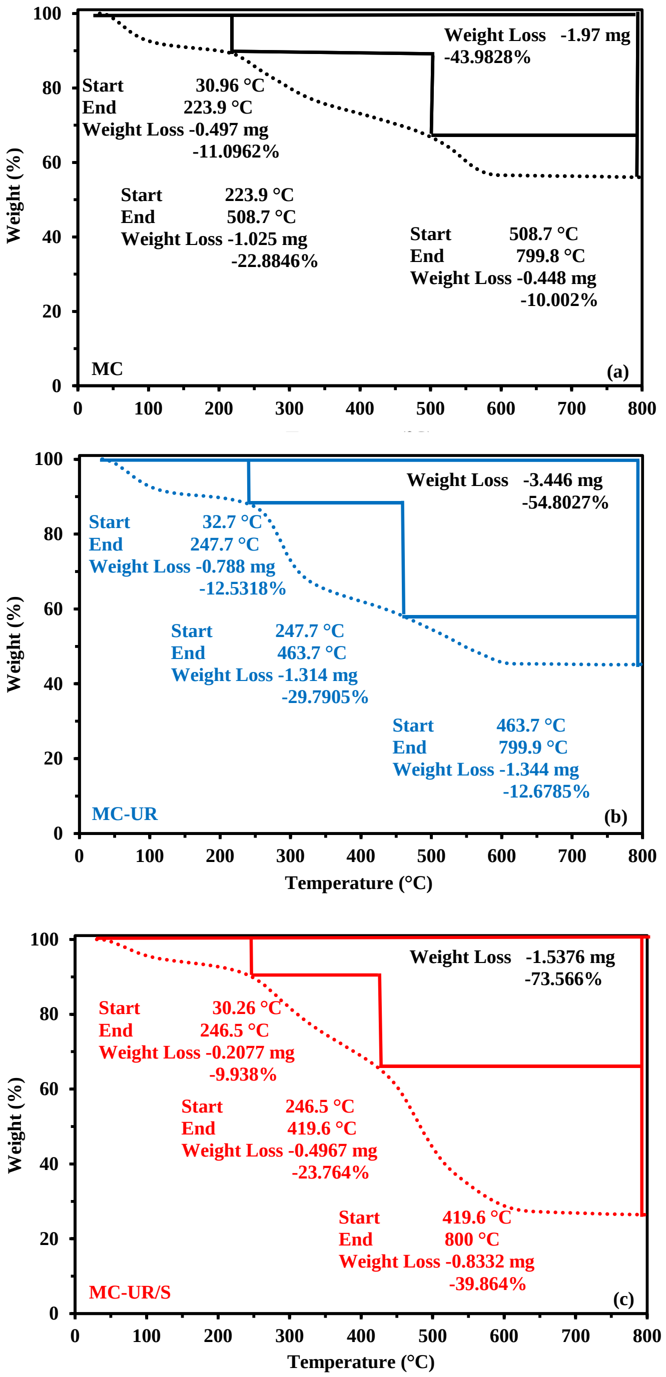

Figure S4. TGA analysis of MC (a), MC-UR (b), and MC-UR/S (c) sorbents.

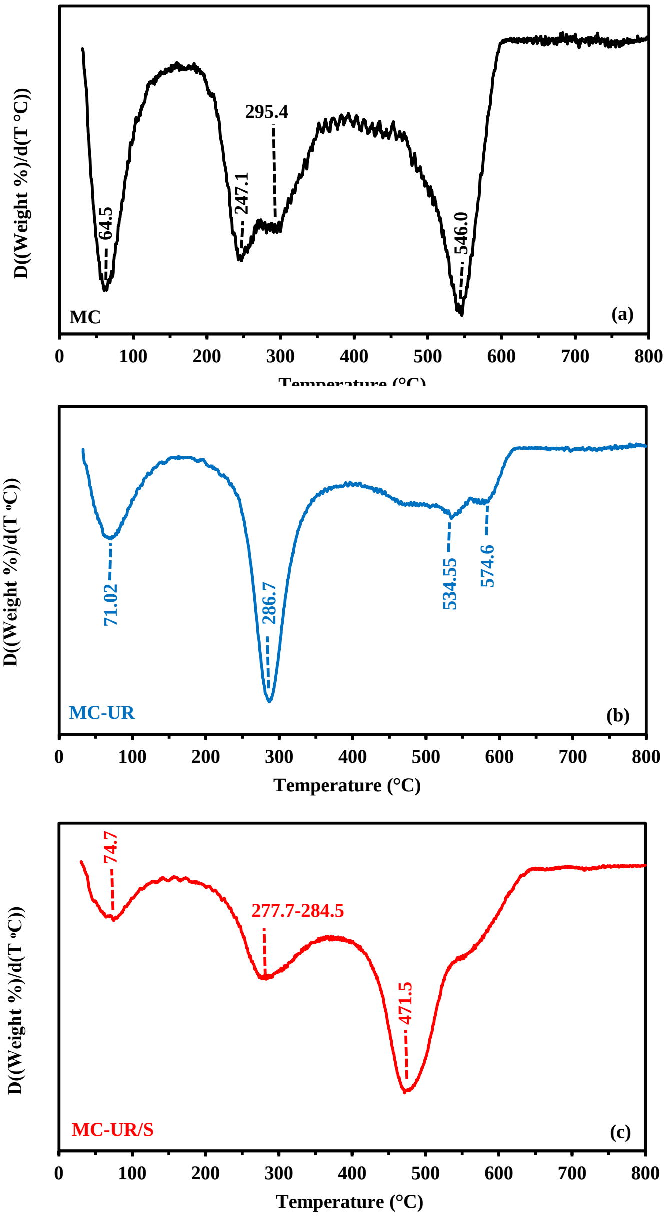

Figure S5. DrTG analysis of MC (a), MC-UR (b), and MC-UR/S (c) sorbents.

Table S2a summarizes the assignments of main FTIR bands for both MC-UR and MC-UR/S at different stages of their utilization (i.e., before and after Ce(III) sorption, and after five cycles of sorption and desorption) (Figure 1). Some typical bands can be identified as the tracers of magnetite fraction: at 633, 590 and 578 cm^{-1}, corresponding to ν(Fe-O, in maghemite and in the tetrahedral site of magnetite). Some typical bands of carbohydrate ring are reported in the region 1350-1000 cm^{-1} (δ(OH), ν(C-O), ν_{as}(C-O-C), and ν(C-O)). The presence of amine and amide groups is proved by a series of bands at 1646-1635 cm^{-1} (δ(NH)), ≈1564 cm^{-1} (δ(NH) in Amide II), 1451 cm^{-1} (δ(NH) in Amide III), ≈1564 cm^{-1} (ν (C-NH) in secondary amine). The urea grafting may be confirmed by the ν(C-O) (in amide and urea; superposed to δ(NH) vibration observed at 1646-1635 cm^{-1}). The sulfonation of MC-UR is characterized by the appearance of the band at 2547 cm^{-1} (ν(SH)), and 1266 cm-^{1} ν(S=O). The broad band at 3360-3450 cm^{-1} is usually associated with the superposition of contributions of ν(OH) and ν(NH). The wavenumber significantly changes with cerium sorption and when the sorbent is analyzed after five cycles of sorption and desorption. The asymmetric and symmetric ν(C-H) vibrations in CH_3 are identified at ≈2960 and ≈2922 cm^{-1}, respectively, while the ν(C-H) vibration in CH_2 appears at 2870-2859 cm^{-1}.

The sorption of Ce(III) is essentially marked by the shifts of the bands δ(NH) (in Amide II) from 1566 to 1549 cm^{-1}, and ν(C-NH) (in secondary amine) from 1382 to 1378 cm^{-1} for MC-UR. The band at 3362 cm^{-1} (overlapping of ν(OH) and ν(NH)) is strongly shifted to 3430 cm^{-1}. Some shifts are also observed for ν(C-O), meaning that both amine and carbonyl groups are involved in Ce(III) sorption. In the case of MC-UR/S, similar changes are also observed; however, in addition, the ν(SH) and ν(S=O) vibration at 2547 and 1266 cm^{-1} disappear or are considerably weakened after Ce(III) binding. The δ(C-N) (in Amide III) is shifted from 1451 to 1461 cm^{-1}. A new band appears at 529 cm^{-1}, which can be assigned to Ce-N vibration. The sorption of Ce(III) onto MC-UR/S involves both amine, carbonyl and sulfonated groups.

The interpretation of these changes in the FTIR spectra may be modulated considering the eventual impact of the pH change (Table S2b). Indeed, the contact of the sorbent with metal solution at pH 5 may cause changes associated with the proper metal binding but also to some alterations of the reactive groups by protonation/deprotonation. For this reason, the spectra of the sorbents before and after Ce(III) sorption are compared with the spectra of the materials exposed to the solutions at target pH (i.e., 5). The most significant changes that can be specifically assigned to the interaction of Ce(III) with MC-UR (Figure S6) concern:

- (a) the broad band at 3430-3370 cm^{-1} (overlapping of ν(OH) and ν(NH)),
- (b) the band at 1549 cm^{-1} δ(NH) (in Amide II), and
- (c) the series of bands between 470 and 400 cm^{-1} (which can be correlated to metal ion-amino bending and metal-nitrogen stretching vibrations [25]).

It is noteworthy that in some spectra a band (more or less marked) may be detected at ≈1720 cm^{-1}; this band may be associated to ν(C=O) and/or δ(sulfonic acid hydrates). This band appears after contact with pH 5 solution for MC-UR (and as a shoulder after U(VI) binding) and systematically in the case of MC-UR/S.

Nitrogen-based reactive groups are significantly affect by pH variation and metal binding. Some additional (and weak) shifts of bands associated with carbonyl groups are also observed but the differentiation with the effects of protonation remain debatable. The differences are more marked in the case of MC-UR/S (Figure S7), where the broad band at 3445-3392 cm^{-1} is also shifted differentially by pH 5 conditioning and by metal sorption. The band at 1266 cm^{-1} (associated with ν(C-O) and ν(S-O), at 1259 cm^{-1} when conditioned at pH 5) is shifted toward 1238 cm^{-1}. The band at 1461 cm^{-1} is also specific to Ce(III)-binding (in relation with δ(C-N) (in Amide III)). Strong changes are also marked by the disappearance of the doublet at 631 and 590 cm^{-1} (appearing in both pH-controlled and raw sorbents), which is replaced with an intense band at 673 cm^{-1}. Mixed contributions of carbonyl/carboxyl, amine and sulfonic contribute to the binding of Ce(III): their environment are affected by the changes (shifts, appearance/disappearance) in their characteristic bands (through the double effect of pH change and direct metal binding).

After metal desorption (at the fifth cycle), the bands are roughly restored; though some bands remained partially shifted or remained strongly weakened (Table S2c). This may be associated to differences imposed by the alternated exposure to neutral and acidic solutions (causing some modifications associated with protonation/deprotonation of reactive groups). Figure S8 compares the FTIR spectra of raw MC-UR to the spectra of the material after the fifth desorption step and after conditioning into 0.2 M HCl solution. The main specific changes associated with recycling are identified at:

- - the broad band at 3362 cm^{-1} (overlapping of ν(OH) and ν(NH)), which is little shifted and enlarged,
- - the bands at 1650-1550 cm^{-1} (amide band),
- - the bands at 1450-1350 cm^{-1} (amine band),
- - the bands at 1250-1050 cm^{-1} (carbohydrate ring and δ(OH)).

These bands, affected by Ce(III) sorption, are partially restored but show significant changes are observed compared with raw sorbent but also for material exposed to 0.2 M HCl solution; this means that the changes are not only due to acidic treatment. The markers of Ce(III) sorption remain altered. This could explain the weak loss in sorption capacity observed during the recycling steps (Section 3.2.5.).

In the case of MC-UR/S, the most significant and specific changes (isolated from the effect of 0.2 M HCl conditioning) concern:

- - the band at 3401 shifted to 3447 cm^{-1} (3419 cm^{-1} for 0.2 M HCl solution),
- - the band at 1451 cm^{-1} (split into two bands at 1461 and 1440 cm^{-1}) (amide band),
- - the band at 1378 cm^{-1} (amine band),
- - the band at 1266 cm^{-1} (shifted to 1251 cm^{-1}) (ν(C-O) and ν(S-O)),
- - the bands 1100-100 cm^{-1} (carbohydrate ring) are shifted toward higher wavenumber.
- - the band at 447 cm^{-1} (which almost disappeared specifically after 5 cycles of reuse, contrary to the simple exposure to 0.2 M HCl).

The cycles of reuse clearly affect the functional groups present on MC-UR/S (through acidic conditioning and alterations directly connected to Ce(III)-interactions), despite the very limited effect of these recycling steps on the sorption performance.

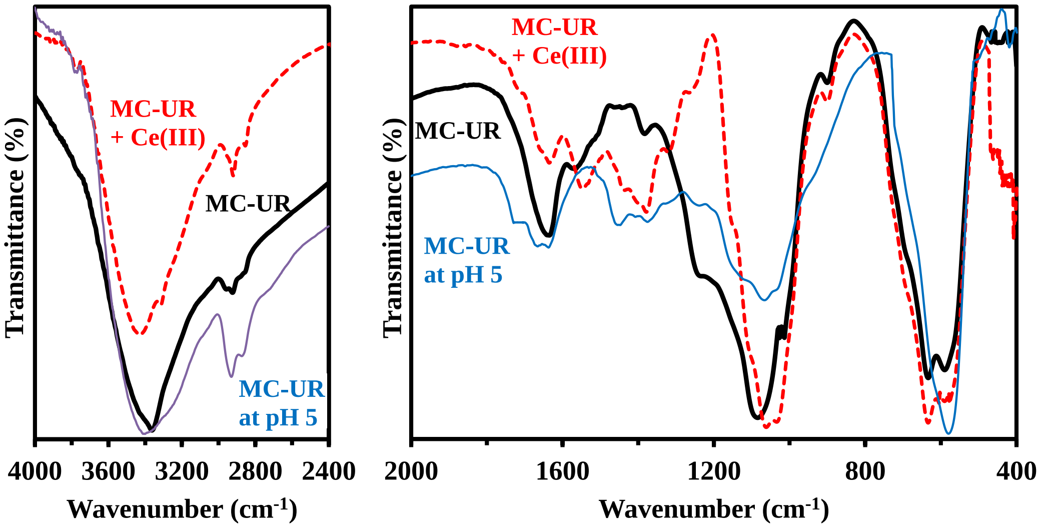

Figure S6. Effect of pH 5 on FTIR spectrum of MC-UR (compared with reference sorbent and Ce-loaded MC-UR).

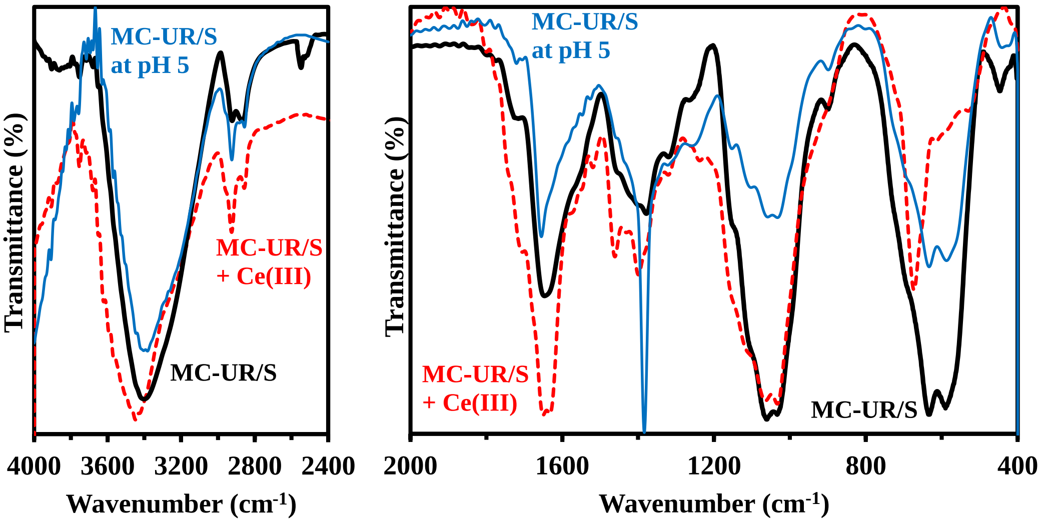

Figure S7. Effect of pH 5 on FTIR spectrum of MC-UR/S (compared with reference sorbent and Ce-loaded MC-UR/S).

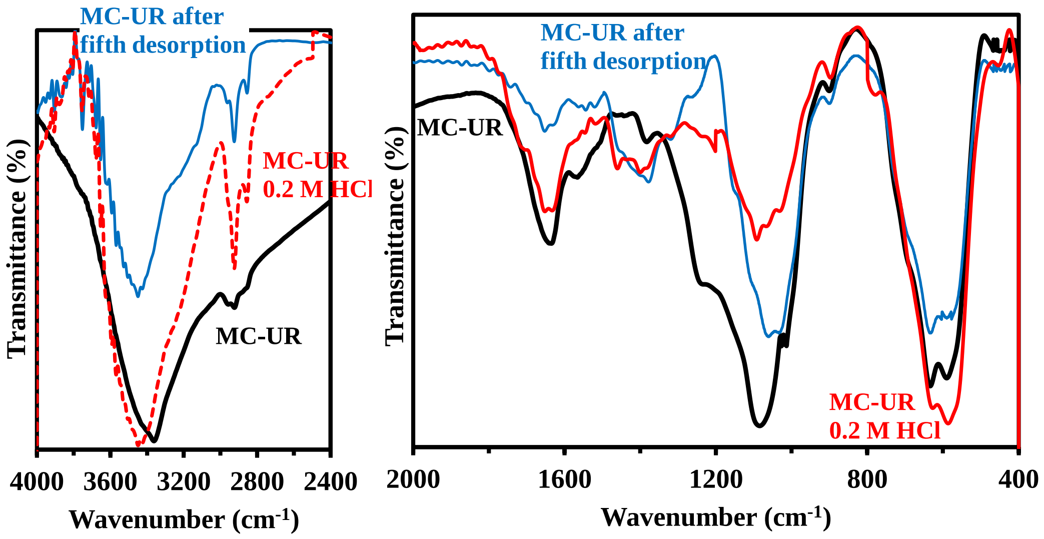

Figure S8. Effect of acidic conditions (0.2 M HCl solution) on FTIR spectrum of MC-UR (compared with reference sorbent and MC-UR after fifth sorption desorption).

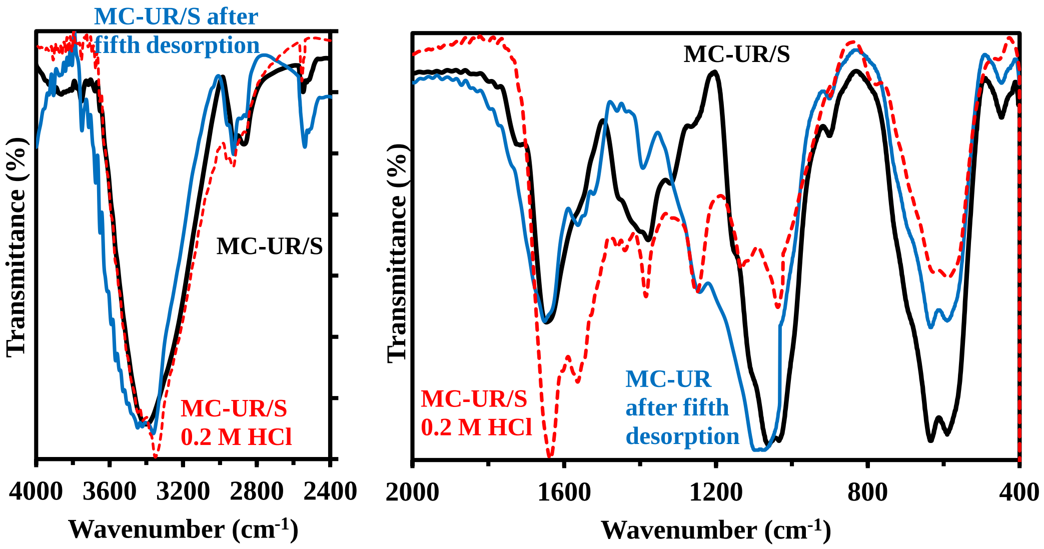

Figure S9. Effect of acidic conditions (0.2 M HCl solution) on FTIR spectrum of MC-UR (compared with reference sorbent and MC-UR after fifth sorption desorption).

Table S2a. FTIR spectra of sorbents (MC-UR, MC-UR/S) before (Raw) and after Ce(III) sorption (+Ce(III)), and after fifth desorption (5th Des.).

Assignment	MC-UR			MC-UR/S			Ref.
	Raw	+Ce(III)	5th Des.	Raw	+Ce(III)	5th Des.	
O-H and N-H str. (overlapping)	3362	3430	3449	3401	3448	3447-3362	[26, 27]
C-H str. (–CH_3) asymm.	2957		2962				[28]
C-H str. (–CH_3) sym.	2924	2920	2924	2922	2925	2925	[28]
C-H str. (-CH_2)	2859	2855	2855	2870	2856	2886	[28]
S-H str.				2547		2537	[29]
C=O str. (amide and urea) / NH bend.	1635	1635	1652	1646	1650<br>1632	1651	[30, 31]
N-H bend. (Amide II)	1566	1549			1520	1564	[26, 32]
-CH_2 bend./C-N bend. (Amide III)				1451	1461		[26, 33]
C-NH str. (2nd amine)	1382	1378	1379	1378	1399	1392	[26]
OH bend.			1320	1320			[34]
C-O str. and S=O str.	1230	1266		1266	1238	1243	[35, 36]
C-O-C str.	1084	1062		1062	1065	1074	[31]
C-O-C asymm. str. and C-O str. oxygen bridge in carbohydrate ring; O=S=O str.	1020-1016	1033	1034	1033	1034	1095	[37, 38]
C-H out-of-plane bend. / -OH str.	900	900	900	900		900	[39]
Fe-O str. (in maghemite) overlapping with C-S bond from thioether (through tautomerization under weak acidic conditions), and C-S str. (shifted by Ce binding associated with tautomerization)	633	633	633	633	673	633	[39]
O-H out-of-plane bend./ Fe-O str.	590	590	590	590		590	[33, 39]
Fe-O str. (tetrahedral site of Fe_3O_4)	578	578	578				[39, 40]
Ce-N vibration					529		[41]
Polysulfides (S-S stretch)				447		447	[35, 36]

Table S2b. FTIR spectra of sorbents (MC-UR and MC-UR/S) before (Raw) and after Ce(III) sorption (+Ce(III)), and after conditioning at pH 5.

Assignment	MC-UR			MC-UR/S			Ref.
	Raw	+Ce(III)	pH 5	Raw	+Ce(III)	pH 5	
O-H and N-H str. (overlapping)	3362	3430	3401	3401	3448	3383	[26]<br>[27]
C-H str. (–CH_3) asymm.	2957						[28]
C-H str. (–CH_3) sym.	2924	2920	2930	2922	2925	2924	[28]
C-H str. (-CH_2)	2859	2855	2873	2870	2856	2855	[28]
S-H str.				2547			[29]
C=O str. (amide and urea) / NH bend.	1635	1635	1666<br>1637	1646	1650<br>1632	1655	[30, 31]
N-H bend. (Amide II)	1566	1549			1520		[26, 32]
-CH_2 bend./C-N bend. (Amide III)			1448	1451	1461		[26, 33]
C-NH str. (2nd amine)	1382	1378	1376	1378	1399	1384	[26]
OH bend.				1320		1322	[34]
C-O str. and S=O str.	1230	1266		1266	1238	1259	[35, 36]
C-O-C str.	1084	1062	1066	1062	1065	1059	[31]
C-O-C asymm. str. and C-O str. oxygen bridge in carbohydrate ring; O=S=O str.	1020<br>1016	1033		1033	1034	1033	[37, 38]
C-H out-of-plane bend. / -OH str.	900	900		900		898	[39]
Fe-O str. (in maghemite) overlapping with C-S bond from thioether (through tautomerization under weak acidic conditions), and C-S str. (shifted by Ce binding associated with tautomerization)	633	633		633	673	633	[39]
O-H out-of-plane bend./ Fe-O str.	590	590		590		587	[33, 39]
Fe-O str. (tetrahedral site of Fe_3O_4)	578	578	580				[39, 40]
Ce-N vibration					529		[41]
Polysulfides (S-S stretch)/MC-UR+Ce(III) from sulfate of Ce salt and from medium (sulfuric acid during pH adj)		441<br>418		447		442<br>424	[35]

Table S2c. FTIR spectra of sorbents (MC-UR and MC-UR/S) before (Raw) and after five cycles of sorption and desorption, and after conditioning with 0.2 M HCl.

Assignment	MC-UR			MC-UR/S			Ref.
	Raw	0.2 M HCl	5th Des.	Raw	0.2 M HCl	5th Des.	
O-H and N-H str. (overlapping)	3362	3425	3449	3401	3419<br>3351	3447<br>3362	[26]<br>[27]
C-H str. (–CH_3) asymm.	2957		2962		2959		[28]
C-H str. (–CH_3) sym.	2924	2925	2924	2922	2927	2925	[28]
C-H str. (-CH_2)	2859	2856	2855	2870		2886	[28]
S-H str.				2547<br>2521	2554	2537<br>2518	[29]
C=O str. (amide and urea) / NH bend.	1635	1633<br>1652	1652	1646	1636	1651	[30, 31]
N-H bend. (Amide II)	1566				1565	1564	[26, 32]
-CH_2 bend./C-N bend. (Amide III)		1460		1451	1461<br>1440		[26, 33]
C-NH str. (2nd amine)	1382	1400	1379	1378	1385	1392	[26]
OH bend.		1321	1320	1320			[34]
C-O str. and S=O str.				1266	1251	1243	[35, 36]
C-O str.	1230	1201					[35]
C-O-C str.	1084	1092<br>1067		1062	1133	1074	[31]
C-O-C asymm. str. and C-O str. oxygen bridge in carbohydrate ring, sym. O=S=O str.	1020<br>1016	1032	1034	1033	1038	1095	[37, 38]
C-H out-of-plane bend. / -OH str.	900	898	900	900	899	900	[39]
Cl (from medium)		778			777		[42]
Fe-O str. (in maghemite) overlapping with C-S bond from thioether (through tautomerization under weak acidic conditions), and C-S str. (shifted by Ce binding associated with tautomerization)	633	628	633	633	627	633	[39]
O-H out-of-plane bend./ Fe-O str.	590	586	590	590	588	590	[33, 39]
Fe-O str. (tetrahedral site of Fe_3O_4)	578		578				[39, 40]

Elemental analysis (summarized in Table S3) confirms the successful functionalization of MC material: with urea grafting, MC-UR shows an increase in nitrogen content that corresponds to a 50% increase in molar content (associated with a little decrease in the relative proportion of O content). The sulfonation of MC-UR (to produce MC-UR/S) produces the appearance of S element, with a molar percentage close to 1.03 mmol S g^{-1}. Compared with N-content in MC-UR, the S/N ratio is close to 4.6; however, compared with the number of amine grafted onto supplementary amine groups of grafted urea, the ratio comes to 1.5. This means that the sulfonation is highly effective, probably associated with the ending amine groups of urea; though complementary sulfonation may also concern other amine groups from chitosan (or bound amine moieties of urea). It is noteworthy that the fraction of iron in the three sorbents remain close to 31-32%; these values cannot be correlated to the residual fractions measured in TGA profiles.

Table S3. Elemental analysis of sorbents.

Sorbent	Composition							
	C (%)	H (%)	O (%)	N (%)	N (mmol g^{-1})	Fe (%)	S (%)	S (mmol g^{-1})
MC	25.98	5.01	32.13	4.42	3.16	32.46	-	
MC-UR	26.36	5.25	30.79	6.58	4.70	31.02	-	
MC-UR/S	18.34	4.89	36.33	6.41	4.58	30.74	3.29	1.03

The pH-drift method allowed evaluating the pH_{PZC} values of MC, MC-UR and MC-UR/S sorbents (Figure S10). The reference material MC shows an intermediary pH_{PZC} value close to 6.23; this is actually very close to the pK_a value of amine groups in chitosan [43] (in the range 6.2-6.7, depending on the degree of acetylation). A similar value (i.e., 6.27) was reported for MC by Hamza et al. [44]. In the case of magnetic crosslinked chitosan associated with MgO, Jawad et al. reported much higher pH_{PZC} value (i.e., close to 9). For magnetic carboxymethyl chitosan aerogel, Lei et al. [23] reported a pH_{PZC} close to 7.3. The grafting of urea slightly increases the pH_{PZC} value up to 6.56; this is associated to the grafting of weakly basic amine groups, while the grafting of acid groups after sulfonation of MC-UR decreases the pH_{PZC} value to 5.40. The functionalization of magnetic chitosan by grafting a substituent bearing carboxylic groups also decreased the pH_{PZC} value [44].

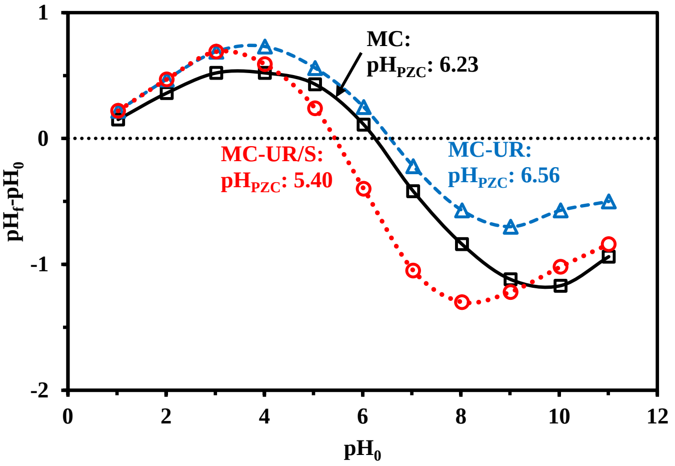

Figure S10. Determination of pH_{PZC} for MC, MC-UR, and MC-UR/S sorbents (pH-drift method – Sorbent dose: 2 g L^{-1}; background salt: 0.1 M NaCl; time: 48 h; agitation: 210 rpm; T: 22 ±1 °C).

Section SC. Sorption properties

SC.1. Effect of pH

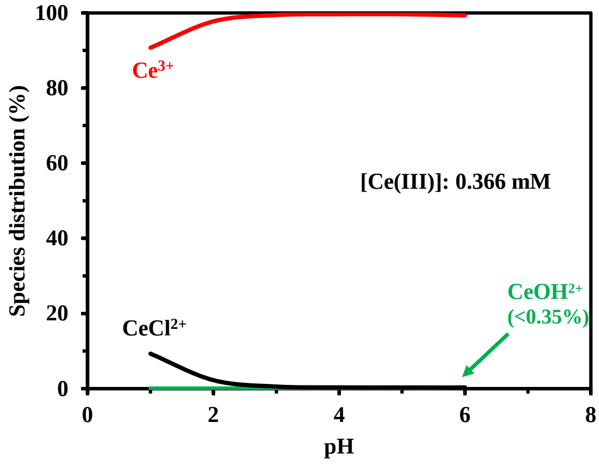

Figure S11. Ce(III) speciation diagram (under experimental conditions of pH study; calculations using Visual Minteq, [45]).

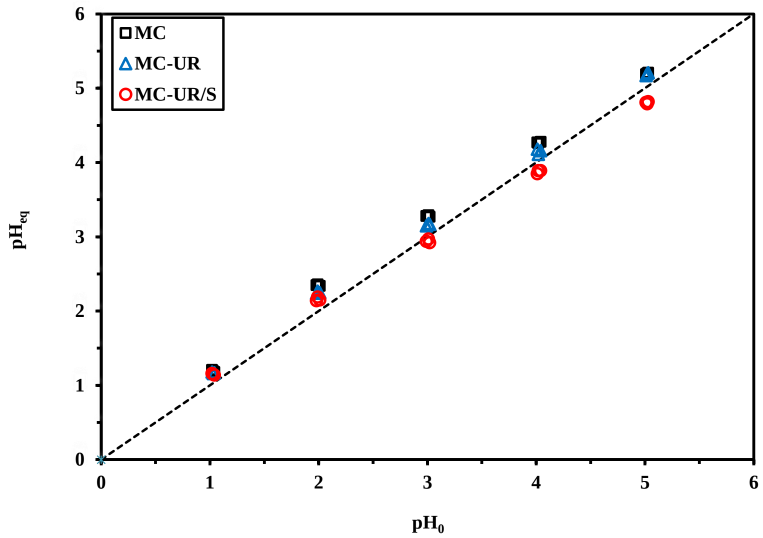

Figure S12. pH variation during Ce(III) sorption using MC, MC-UR, and MC-UR/S sorbents (Sorbent dose, SD: 400 mg L^{-1}; C_0: 0.366 mmol Ce L^{-1}; time: 48 h; v: 210 rpm; T: 22 ±1 °C).

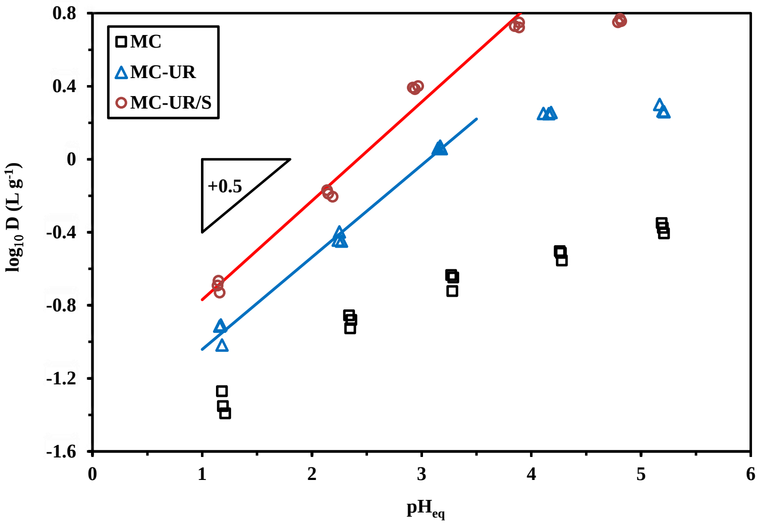

Figure S13. Distribution ratio as a function of pH_{eq} (log_{10} plot) for Ce(III) sorption using MC, MC-UR, and MC-UR/S sorbents (Sorbent dose, SD: 400 mg L^{-1}; C_0: 0.366 mmol Ce L^{-1}; time: 48 h; v: 210 rpm; T: 22 ±1 °C)

SC.2. Uptake kinetics

Table S4. Uptake kinetics for Ce(III) sorption using MC, MC-UR, and MC-UR/S sorbents – Parameters of the models.

Sorbent			MC			MC-UR			MC-UR/S		
Model	Parameter	Run	#1	#2	#3	#1	#2	#3	#1	#2	#3
Exper.	$q_{eq,exp}$		0.274	0.283	0.303	0.853	0.830	0.848	1.23	1.26	1.21
PFORE	$q_{eq,1}$		0.288	0.314	0.328	0.907	0.887	0.900	1.31	1.34	1.28
	$k_1 \times 10^2$		4.13	2.49	2.70	3.24	3.33	3.21	3.73	3.78	3.92
	R^2		0.982	0.912	0.940	0.975	0.968	0.979	0.963	0.967	0.963
	AIC		-141	-114	-121	-103	-99	-107	-86	-87	-85
PSORE	$q_{eq,2}$		0.344	0.414	0.420	1.13	1.10	1.12	1.60	1.64	1.56
	$k^2 \times 10^2$		12.2	5.23	5.96	2.85	3.01	2.88	2.42	2.41	2.66
	R^2		0.955	0.883	0916	0.950	0.940	0.955	0.931	0.935	0.929
	AIC		-127	-110	-116	-95	-91	-98	-78	-79	-77
RIDE	$D_e \times 10^{14}$		7.85	4.93	5.18	4.46	4.64	4.39	3.71	3.64	3.71
	R^2		0.962	0.880	0.911	0.948	0.938	0.951	0.926	0.930	0.924
	AIC		-127	-109	-115	-89	-86	-92	-73	-73	-72

Units: q, mmol L^{-1}; k_1, min^{-1}; k_2, g $mmol^{-1}$ min^{-1}; D_e, m^2 min^{-1}.

SC.3. Sorption isotherms

Table S5. Ce(III) sorption isotherm using MC – Parameters of the models.

Model	Parameters	#1	#2	#3	Cumulated
Experimental	$q_{m,exp.}$	0.495	0.541	0.549	0.549
Langmuir	$q_{m,L}$	0.587	0.636	0.654	0.625
	b_L	1.727	1.389	1.402	1.498
	R^2	0.982	0.982	0.965	0.968
	AIC	-76	-75	-67	-209
Freundlich	k_F	0.335	0.336	0.347	0.339
	n_F	2.57	2.39	2.41	2.45
	R^2	0.963	0.973	0.955	0.953
	AIC	-69	-71	-65	-198
Sips	$q_{m,S}$	0.656	0.782	0.799	0.740
	b_S	1.246	0.850	0.869	0.974
	n_S	1.199	1.304	1.300	1.267
	R^2	0.983	0.984	0.967	0.970
	AIC	-74	-73	-64	-211
Temkin	A_T	26.45	24.05	24.80	25.05
	b_T	21.87	20.86	20.41	21.02
	R^2	0.978	0.973	0.955	0.959
	AIC	-74	-71	-65	-176
Dubinin-Radushkevich	$q_{m,DR}$	0.484	0.511	0.528	0.507
	$\beta_{DR} \times 10^8$	6.63	8.59	8.65	7.88
	E_{DR}	3.88	3.41	3.40	3.56
	R^2	0.953	0.953	0.938	0.948
	AIC	-65	-62	-59	-179

Units – q, mmol g^{-1}; b_L, L $mmol^{-1}$; n, dimensionless; k_F, $mmol^{1-1/n_F}$ L^{-1/n_F} g^{-1}; b_S: $(mmol\ L^{-1})^{n_S}$; A_T, L mol^{-1}; b_T, J kg $mmol^{-2}$; β_{DR}: mol^2 kJ^{-2}; E_{DR}: k2.275 mmol Ce g^{-1} for MC-UR/S.J mol^{-1}.

Table S6. Ce(III) sorption isotherm using MC-UR – Parameters of the models.

Model	Parameters	#1	#2	#3	Cumulated
Experimental	$q_{m,exp.}$	1.319	1.278	1.328	1.328
Langmuir	$q_{m,L}$	1.337	1.289	1.290	1.305
	b_L	4.969	5.752	6.730	5.743
	R^2	0.978	0.977	0.971	0.968
	AIC	-53	-53	-50	-155
Freundlich	k_F	0.992	0.978	1.00	0.990
	n_F	3.36	3.45	3.53	6.45
	R^2	0.952	0.953	0.950	0.940
	AIC	-45	-46	-44	-135
Sips	$q_{m,S}$	1.482	1.450	1.462	1.470
	b_S	2.746	2.813	2.973	2.796
	n_S	1.292	1.334	1.361	1.338
	R^2	0.981	0.982	0.977	0.974
	AIC	-51	-52	-49	-160
Temkin	A_T	92.02	100.5	111.8	101.2
	b_T	10.51	10.88	10.90	10.77
	R^2	0.978	0.982	0.979	0.974
	AIC	-54	-56	-54	-162
Dubinin-Radushkevich	$q_{m,DR}$	1.227	1.197	1.212	1.211
	$\beta_{DR} \times 10^8$	2.794	2.506	2.284	2.505
	E_{DR}	5.98	6.32	6.62	6.32
	R^2	0.967	0.967	0.963	0.956
	AIC	-48	-49	-48	-145

Units – q, mmol g^{-1}; b_L, L $mmol^{-1}$; n, dimensionless; k_F, $mmol^{1-1/n_F}$ L^{-1/n_F} g^{-1}; b_S: $(mmol\ L^{-1})^{n_S}$; A_T, L mol^{-1}; b_T, J kg $mmol^{-2}$; β_{DR}: mol^2 kJ^{-2}; E_{DR}: kJ mol^{-1}.

Table S7. Ce(III) sorption isotherm using MC-UR/S – Parameters of the models.

Model	Parameters	#1	#2	#3	Cumulated
Experimental	$q_{m,exp.}$	2.132	2.176	2.175	2.176
Langmuir	$q_{m,L}$	2.253	2.293	2.280	2.275
	b_L	5.482	5.259	5.395	5.375
	R^2	0.995	0.995	0.992	0.992
	AIC	-57	-56	-52	-163
Freundlich	k_F	1.702	1.721	1.723	1.715
	n_F	3.163	3.121	3.160	3.15
	R^2	0.937	0.936	0.946	0.929
	AIC	-29	-29	-31	-93
Sips	$q_{m,S}$	2.235	2.260	2.361	2.284
	b_S	5.841	5.918	4.229	5.215
	n_S	0.977	0.957	1.100	1.012
	R^2	0.995	0.995	0.993	0.992
	AIC	-53	-52	-49	-160
Temkin	A_T	72.11	68.19	79.76	73.26
	b_T	5.78	5.64	5.85	5.76
	R^2	0.992	0.991	0.993	0.990
	AIC	-52	-51	-54	-155
Dubinin-Radushkevich	$q_{m,DR}$	2.091	2.122	2.101	2.105
	$\beta_{DR} \times 10^8$	2.747	2.834	2.667	2.754
	E_{DR}	6.03	5.94	6.12	6.03
	R^2	0.993	0.993	0.986	0.989
	AIC	-54	-53	-46	-151

Units – q, mmol g^{-1}; b_L, L $mmol^{-1}$; n, dimensionless; k_F, $mmol^{1-1/n_F}$ L^{-1/n_F} g^{-1}; b_S: $(mmol\ L^{-1})^{n_S}$; A_T, L mol^{-1}; b_T, J kg $mmol^{-2}$; β_{DR}: mol^2 kJ^{-2}; E_{DR}: kJ mol^{-1}.

Table S8. Comparison of Ce(III) sorption properties with alternative sorbents.

Sorbent	pH	Time	$q_{m,exp}$	$q_{m,L}$	b_L	Ref.
Poly(acrylamide/itaconic acid/TiO_2)	6	60	-	0.527	13.3	[46]
Phosphonomethyl iminodiacetic/PAN MOF	4	20	2.08	1.61	3.10	[47]
Magnetic nanocellulose	5	360	-	2.52	0.154	[48]
Titanium phosphate	7	180		1.13	1.68	[49]
Titanosilicate ETS-10	3	7	0.50	1.16	1.12	[50]
Sulfonated ALPEI beads	5	40	0.61	0.71	2.35	[3]
Polystyrene/poly(hydroxamic acid) copolymer	1	240	2.20	2.59	0.635	[7]
HKUST-1 MOF	6	360	-	2.47	9.95	[51]
Zirconium titanate/PAN	4.3	1440	-	0.0076	11.8	[52]
Spirulina biomass	5-5.5	180	-	0.272	0.841	[53]
Carboxymethylcellulose HIPE	5	30	2.39	2.38	0.520	[54]
Amino-phosphonic acid/activated carbon	6	240	-	0.673	0.222	[55]
Grapefruit peel	5	60	-	1.14	5.28	[56]
Tangerine peel	5	60	-	1.16	16.1	[57]
Turbinaria conoides	4.9	150	1.06	1.09	4.90	[58]
Crab shell	6	120	1.01	1.03	6.03	[59]
Platanum orientalis leaf	4	60	-	0.229	21.0	[60]
MC	5	60	0.549	0.625	1.50	This work
MC-UR	5	60	1.33	1.31	5.74	This work
MC-UR/S	5	60	2.18	2.28	5.38	This work

Units – Time, min; q_m, mmol Ce g^{-1}; b_L, L $mmol^{-1}$.

SC.4. Selectivity tests

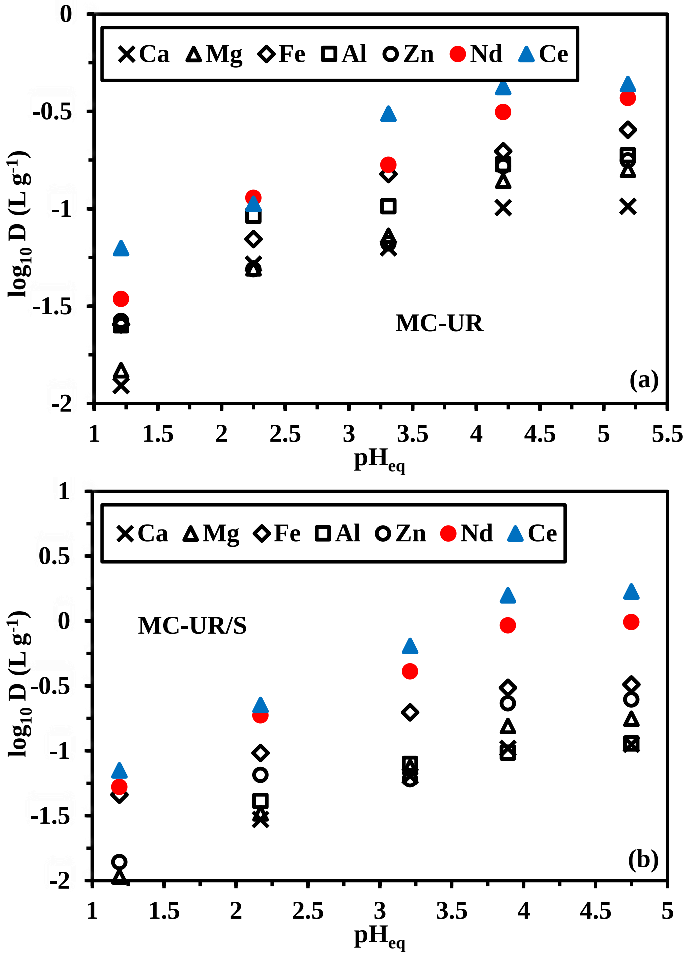

Figure S14. Effect of pH_{eq} on the distribution ratio (log_{10} unit) using MC-UR (a) and MC-UR/S (b) sorbents from sorption tests on multicomponent equimolar solutions (SD: 1 g L^{-1}; C_0: ≈ 1 mmol L^{-1}; time: 10 h; v: 210 rpm; T: 22 ±1 °C).

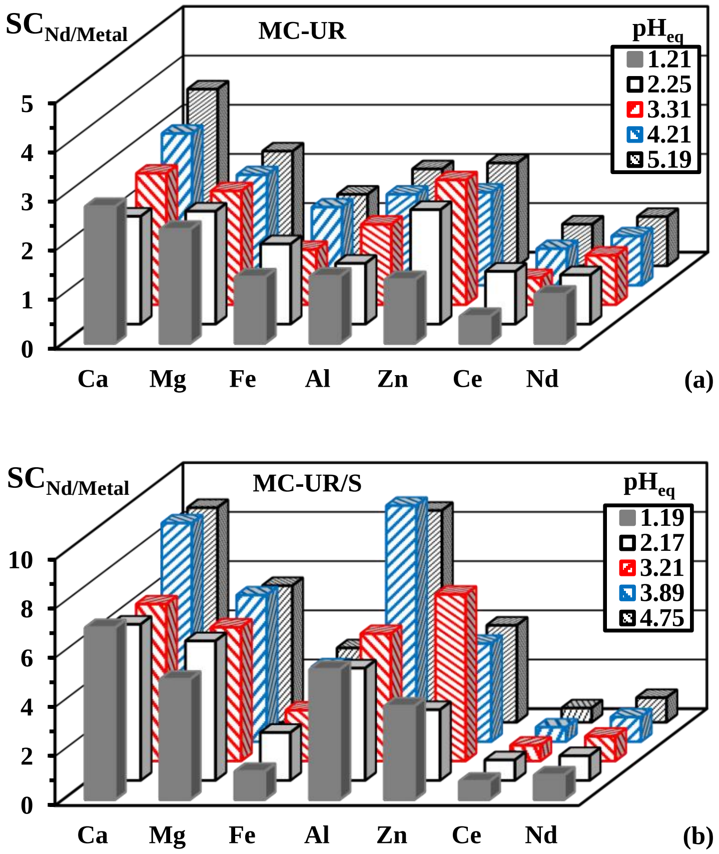

Figure S15. Selectivity tests for Nd(III) sorption using MC-UR (a) and MC-UR/S (b) sorbents from multicomponent equimolar solutions – effect of pH_{eq} on $SC_{Nd/metal}$ (SD: 1 g L^{-1}; C_0: ≈ 1 mmol L^{-1}; time: 10 h; v: 210 rpm; T: 22 ±1 °C).

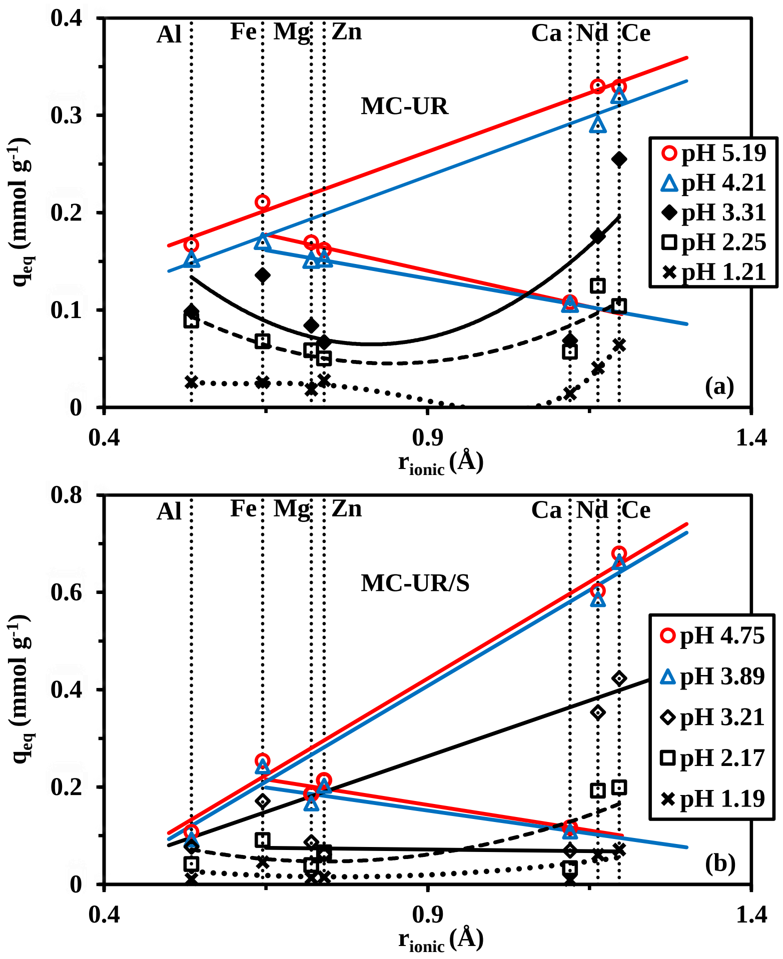

Figure S16. Correlation of sorption with Shannon ionic radius for metal sorption in multicomponent solutions at different pH values (SD: 1 g L^{-1}; C_0: ≈ 1 mmol L^{-1}; time: 10 h; v: 210 rpm; T: 22 ±1 °C).

At the highest pH values (i.e., pH 3.9-5.2): the sorption of trivalent metal ions is linearly correlated (and increases) with the ionic radius, while the sorption follow the reciprocal trend for divalent cations (in the case of MC-UR/S, the same trend is roughly followed for pH 3.21).

At the lowest pH values (i.e., pH 3.3-1.2): the linear trends cannot be detected (spline interpolation, order 2 or more). These differences are probably associated with the sorption mechanisms induced by site protonation (decreasing, in acidic solution, the contribution of chelation mechanism). The shift between MC-UR and MC-UR/S regarding the limit pH (lower for MC-UR/S) is consistent with the acid-base properties (pH_{PZC}) of the sorbents. At high pH, the covalent mechanisms is more representative of binding mechanisms than are ionic-exchange reactions.

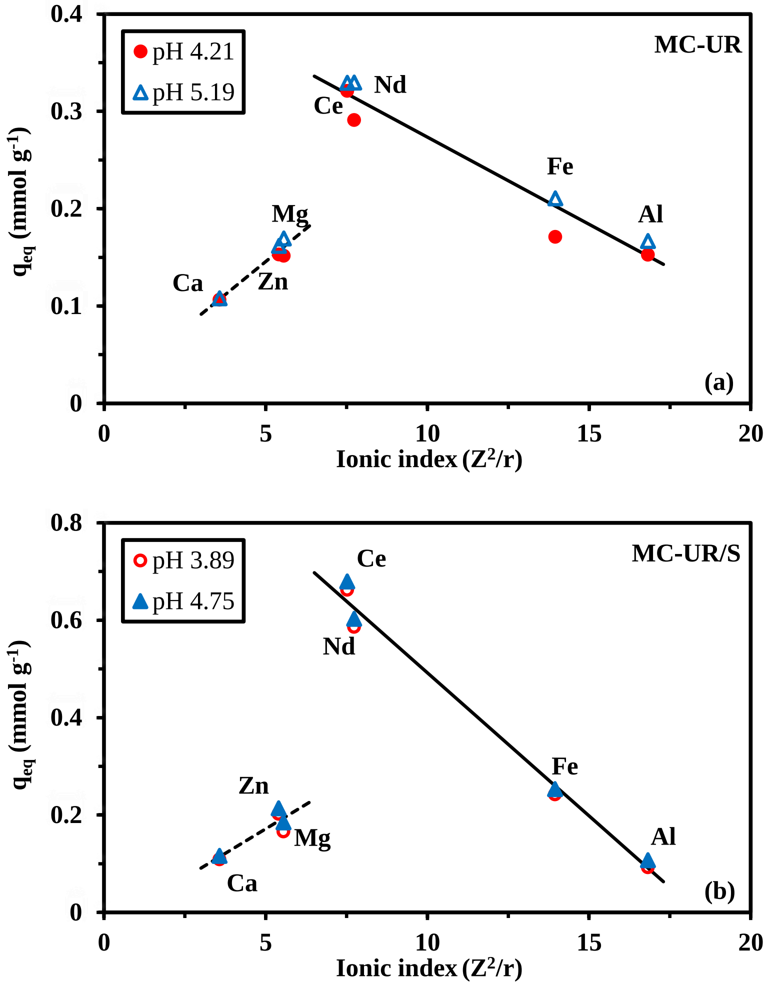

Figure S17. Correlations between sorption capacities and ionic index for the sorption of metal ions from multi-component equimolar solutions using MC-UR (a) and MC-UR/S (b).

SC.5. Desorption tests

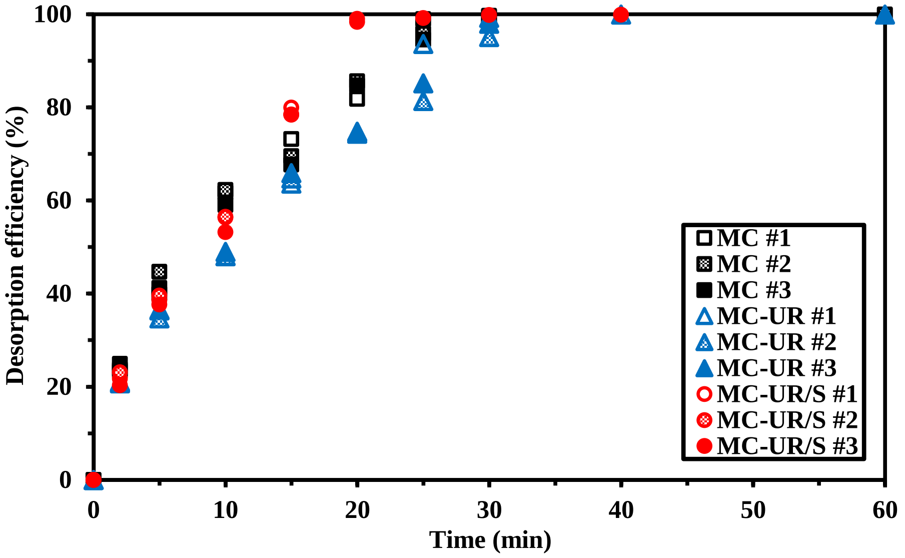

Figure S18. Ce(III) desorption form metal-loaded MC, MC-UR, and MC-UR/S sorbents (samples collected from uptake kinetics; desorption: SD: 1.6 g L^{-1}; Eluent: 0.2 M HCl; v: 210 rpm; T: 22 ±1 °C).

Section SD. Application to ore treatment

SD.1. Location and Area

Ramlet Hemeyir area lies in southwestern Sinai, about 72 km, to the southeast of Abu Zenima city. It is located between latitudes 29⁰ 0/ - 29⁰ 2/ N and longitudes 33⁰ 30/ – 33⁰ 33/ E (Figure S19). The Adedia Formation in the study area is up to 72 m thick. The uppermost beds of this formation are highly ferruginous and usually stained by manganese and iron oxyhydroxides. The studied sample consists of white to pale brownish pebbly and ferruginous sandstones from the topmost of El Adedia Formation.

The top of Adediya Formation (1-2 m thick) is consisting of ferruginous sandstone and ferruginous siltstone soil and shows signs of lateritization mainly of iron and less alumina. The top of this section is of red ferruginous clay. This horizon represents the unconformity between the Adediya Formation and the overlying Um Bogma Formation. The lithostratigraphy of Adediya Formation was studied in several localities at southwestern Sinai named, G. Adediya, G. Sarabit El Khadim, Abu Hamata, El Dakran and W. El Khaboba. There are important radioactive exposures in G. Um Hamd, W. El Sahu and in G. Abu Trifia to the southwest of G. Adediya. In these two localities the Adediya Formation is reached to 60 m in thickness and is consisted of sandstone, fine to medium grained, partially pebbly with distinguished tabular and convolute cross bedding. Sometimes it becomes yellow, brown and red due to staining with iron oxides.

Several phosphate minerals were identified from the heavy fractions of these ferruginous clastics. These minerals are xenotime (YPO_4), wavellite $Al_3(PO_4)_2(OH)_2.5H_2O$, saleeite $Mg(UO_2)_2(PO_4)_2.H_2O$ and vivianite $Fe_3(PO_4)_2.8H_2O$. Others are oxides as becqurelite ($CaU_6O_{19}.11H_2O$), euxenite $(Y,Ca,Ce,U,Th)(Nb,Ta,Ti)_2O_6$, rutile ($TiO_2$), ilmenite ($FeTiO_3$), quartz ($SiO_2$), goethite ($\alpha$FeO)(OH) and hematite ($Fe_2O_3$). The silicate minerals are mainly zircon ($ZrSiO_4$) and less dominating allanite $(Ce,Ca,Y)(Al,Fe)_3(SiO_4)_3(OH)$ and kaolinite $(Al_2Si_2O_5)(OH)_4$, with carbonate mineral calcite ($CaCO_3$). These minerals reflect the supergene processes which had been happened at the top of El Adedia Formation. Table S9 summarizes the main metal (and Si) present in the ore sample.

Table S9. Composition of the ore.

Major oxides	Weight percentage (%)	Trace elements	Concentration (ppm)
SiO_2	40	U	2600
TiO_2	0.33	Ni	128
Al_2O_3	3.4	Pb	900
Fe_2O_3	26	B	200
CaO	1.2	Cu	165
MgO	3.2	V	150
MnO	1.8	Zr	180
Na_2O	0.9	Th	60
K_2O	0.7		
P_2O_5	3.6	LOI	4.06
Re_2O_3	12.6		

SD.2. Treatment of ore

The ore was leached using sulfuric acid solution (200 g H_2SO_4 L^{-1}) at 150 °C for 2 h. The S/L ratio was set to 1/3. Factually, 1 kg of ore material (grinded at 1 cm average size) was maintained under agitation with 3 L of leaching agent. The composition of the leachate (2280 mL) is reported in Table S10.

This leachate contains high concentrations of Fe(III) and Al(III) (20.8 g L^{-1} and 8.17 g L^{-1}, respectively) that may interfere in the sorption process and the valorization of target and valuable metals (i.e., REE(III) and U(VI)). A series of pre-treatments was operated in order to improve the separation of these metals. The first step consisted of processing the leachate by sorption onto Amberlite IRA-400) at pH 1.8 ±0.2 for recovering

uranyl ions: the leachate (1 L) was pumped through a fixed-bed column containing 100 g of resin (flow rate of 1 mL min^{-1}). Uranium recovery reached up to 80% (see Table S10). The second step in the process consisted of REE recovery using a cationic ion-exchange resin (i.e., Dowex 20 X8) in a fixed-bed column (200 g); the pH of the solution was adjusted to 4 before feeding the column for iron precipitation, other metals ion were co-precipitated. Table S10 also reports the variation in the concentration of selected metal ions at this step.

Table S10. Pre-treatment of ore leachate – Concentrations (mg L^{-1}) of selected metals.

Metal ion	Leachate	Outlet of Amberlite IRA-400	Residue after pH 4 control	Outlet of Dowex 50 X8	Residue after pH 5 control
Si(IV)	128	101.7	96.94	92.18	86.6
Al(III)	8170	7695	5439	4998	156.8
Fe(III)	20800	19886	233.5	196.8	148.3
Ca(II)	1170	1068	795.9	736.2	595.7
Mn(II)	980	886	554.2	521.6	347.8
Ni(II)	60.8	57.47	52.18	47.8	48.9
Cu(II)	31.5	29.53	26.95	25.02	12.6
REE(III)	8700	8280	7895	498.6	210.7
Nd (III)	980	876	803.1	102.7	57.4
Ce(III)	430	412	386.5	58.8	32.1
U(VI)	200	37.9	33.1	30.8	29.7
Pb(II)	80.31	77.4	69.8	52.8	43.2

The raffinate (residue after this series of treatment) was used for testing MC-UR/S sorbent. The sorbent dose was set to 2 g L^{-1}; the suspension was agitated for 10 h (v: 210 rpm, T: 21 ±1 °C). The pH of the raffinate was controlled to 5 different pH values (in the range 1-5). Residual concentrations (and sorption efficiencies) are reported in Table S11.

Table S11. Composition of ore leachate, raffinate (including metal recovery efficiency, RE (%)), and residue of adsorption onto MC-UR/S at different pH_{eq} values.

Metal ion	Leachate	Raffinate		Sorption									
				pH_{eq} 1.13		pH_{eq} 2.19		pH_{eq} 3.13		pH_{eq} 4.15		pH_{eq} 4.87	
		C_R	RE	C_{eq}	SE	C_{eq}	SE	C_{eq}	SE	C_e	SE	C_{eq}	SE
Si(IV)	128	86.6	32.34	85.9	0.84	85.2	1.61	85.0	1.89	84.1	2.94	83.5	3.59
Al(III)	8170	156.8	**98.08**	149.8	4.46	142.7	8.99	136.9	12.69	132.5	15.50	128.2	18.24
Fe(III)	20800	148.3	**99.29**	139.8	5.70	138.2	6.81	122.9	17.15	102.7	30.75	99.8	**32.73**
Ca(II)	1170	595.7	49.09	591.3	0.74	585.8	1.66	579.6	2.70	572.9	3.83	570.8	4.18
Mn(II)	980	347.8	64.51	332.8	4.31	329.5	5.26	318.9	8.31	311.6	10.41	308.2	11.39
Ni(II)	60.8	48.9	19.57	46.6	4.74	45.1	7.77	43.9	10.29	40.1	17.98	39.0	20.29
Nd (III)	980	57.4	**94.14**	54.0	5.96	47.4	17.47	41.8	27.13	37.0	35.59	35.2	**38.71**
Ce(III)	430	32.1	**92.53**	30.6	4.67	25.0	22.21	19.0	40.84	13.1	59.28	13.0	**59.38**
U(VI)	200	29.7	85.15	29.1	2.02	27.7	6.84	24.4	17.78	21.1	28.96	19.7	**33.60**
Pb(II)	80.31	43.2	46.21	42.2	2.36	42.0	2.80	38.2	11.64	35.9	16.99	32.4	25.05
REE(III)	8700	210.7	**97.58**	198.7	5.70	160.7	23.74	124.4	40.97	92.3	56.19	83.4	**60.42**

Units – C, mg L^{-1}.; RE, % (Experimental conditions for sorption tests– SD: 2 g L^{-1}; T: 22 ±1 °C; time: 10 h; v: 210 rpm).

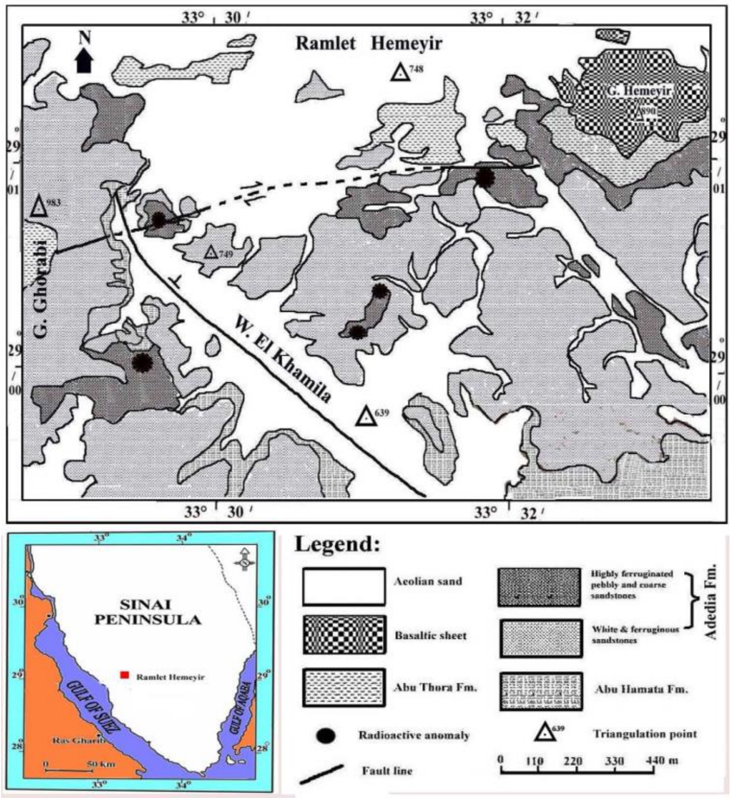

Figure S19. Geological map of the studied area.

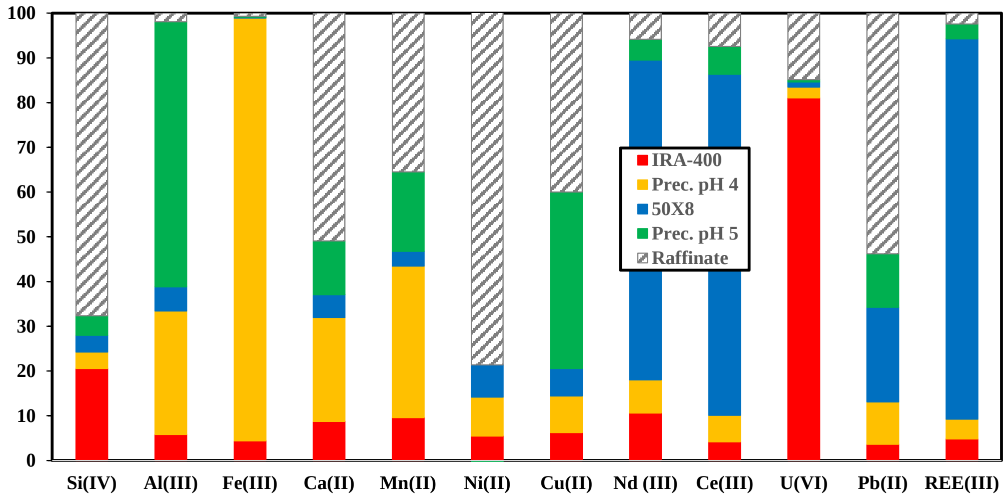

Figure S20. Distribution of main elements in the different compartments of the pre-treatment of leachates.

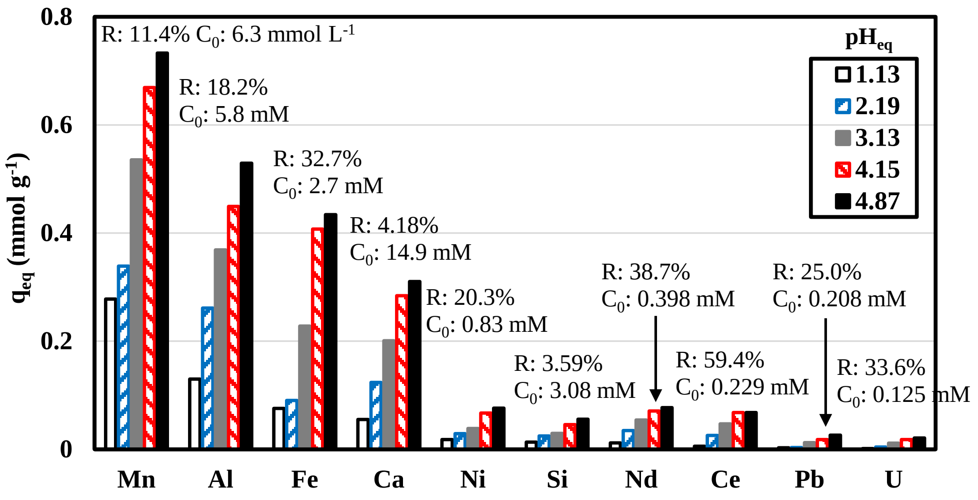

Figure S21. Effect of pH on metal recovery on sorption capacity for selected elements from ore raffinate using MC-UR/S – Sorption capacities (q_{eq}), maximum sorption efficiency (R(%), at optimum pH_{eq}, i.e., 4.87), and reference initial metal concentration in the raffinate (C_0, mmol L^{-1}) (SD: 2 g L^{-1}; T: 22 ±1 °C; time: 10 h; v: 210 rpm).

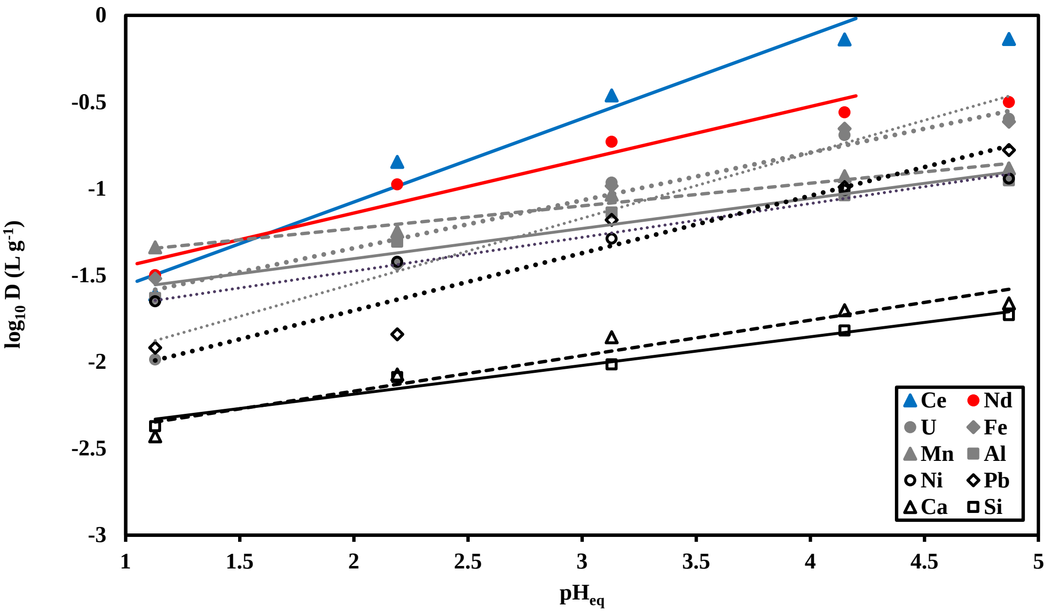

Figure S22. Metal recovery from ore raffinate using MC-UR/S sorbent – Effect of pH_{eq} on the distribution ratio (log_{10} plot).

Metal	Si	Ca	U	Fe	Pb	Al	Ce	Nd	Mn	Ni
Slope	0.165	0.205	0.377	0.276	0.331	0.174	0.394	0.259	0.131	0.195
R^2	0.973	0.941	0.957	0.943	0.935	0.940	0.912	0.914	0.966	0.987
Slope*							0.481	0.308		
R^2							0.961	0.940		

*: breakdown of the linear trend at pH > 4.15 – recalculation with data pH_{eq} 1.17 to 4.15.

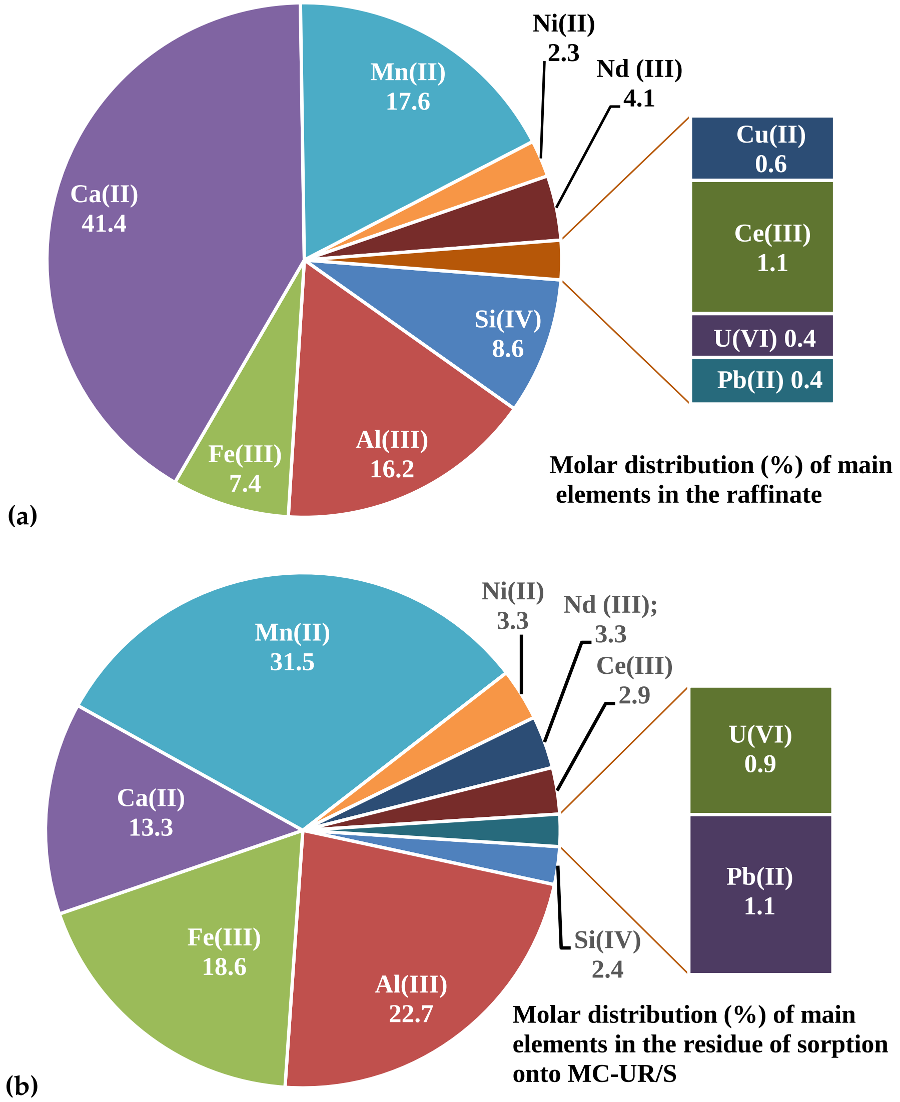

Figure S23. Molar distribution of main elements in the raffinate (a) and the residue of sorption step at pH_{eq} 4.87 (b) (numbers: actual molar percentages of target elements in the raffinate; SD: 2 g L-1; v: 210 rpm; T: 21 ±1 °C; time: 10 h).

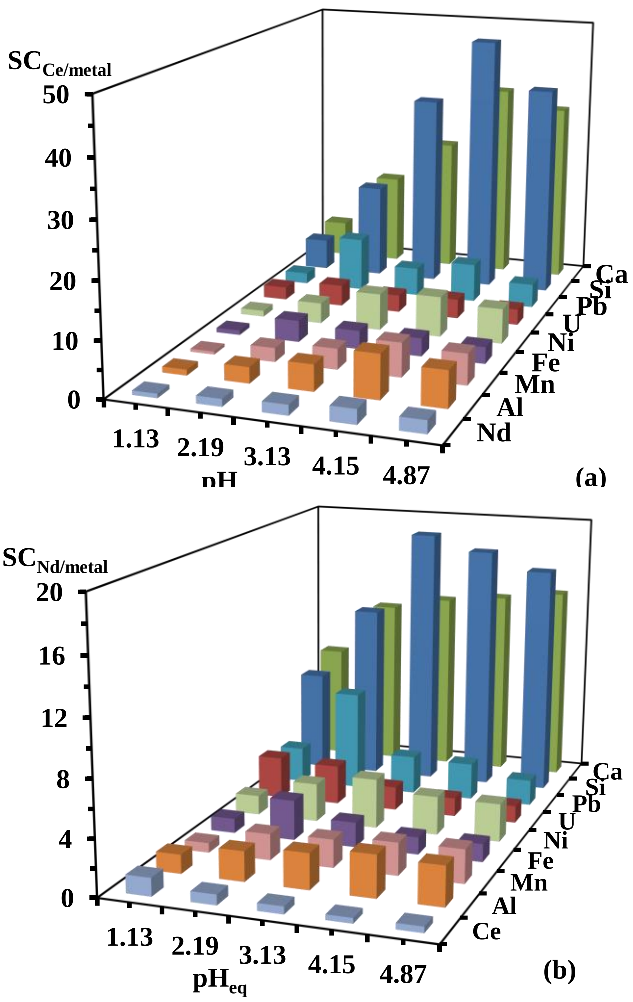

Figure S24. Effect of pH on the selectivity coefficients for metal sorption from ore raffinate – (a) $SC_{Ce/metal}$ and (b) $SC_{Nd/metal}$ (SD: 2 g L^{-1}; T: 22 ±1 °C; time: 10 h; v: 210 rpm).

Section SE. Synthesis of sorbents

SE.1. Preparation of magnetite nanoparticles.

Thermally co-precipitation method was used to obtain the magnetite nano particles, which known as Massart method [61]. This was performed by dissolution of mixture of hydrated ferrous sulfate ($FeSO_4.7H_2O$; 5.0 g) and ammonium ferric sulfate ($(NH_4)Fe(SO_4)_2.12H_2O$; 17.35 g) in the water medium. The reaction was maintained at 40-50 °C for 60 min (under vigorous stirring); the magnetite (precipitation) was performed by pH control to 10-12 using 5 M NaOH solution, while the stirring continued for 5 h at 45 °C. The magnetite nanoparticles were magnetically separated, washed with demineralized water/acetone, and dried at 50 °C for 20 h (≈4.4 g, d.w.).

SE.2. Preparation of magnetite chitosan microparticles (MC)

Two grams of chitosan particles were dissolved in 50 mL acetic acid (AA) solution (7%, w/w). Magnetite particles (2 g) added to the mixture and refluxed for 7 h at 70 °C. Addition of 10 mL glutaraldehyde solution and the reaction maintain stirring for further 2 h at 50 °C. The produced composites were collected from the solution using magnetic bar, washed with water and acetone before dried at 50 °C overnight to produce MC (≈5.71 g, d.w.).

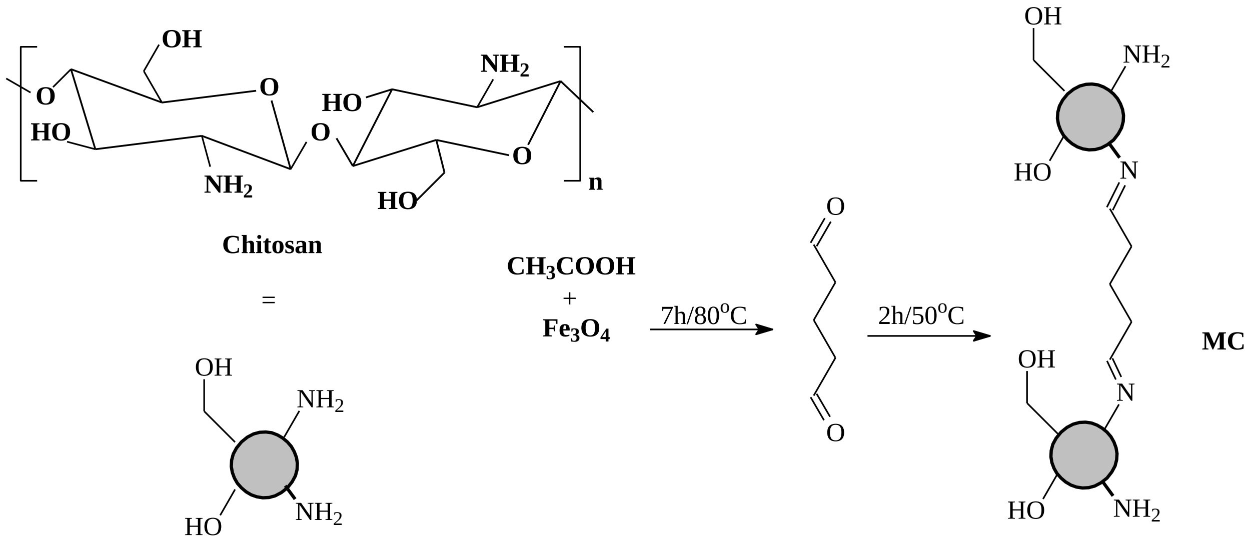

SE.3.. Functionalization of magnetic chitosan (MC) microparticles: MC-UR and MC-UR/S

Four grams of urea was dissolved in 100 mL distilled water, after dissolution, 10 g of magnetite was added to the solution with continuous stirring for 30 min. Twenty mL of formaldehyde solution was added. The reaction was maintained at 50 °C for 2 h. This mixture was added to the chitosan solution (2 g dissolved in a solution of 50 mL acetic acid solution, 7%, w/w). The mixture was refluxed at 70 °C for 7 h. After cooling of the solution, a solution of glutaraldehyde (10 mL) was added to the mixture for enhancing the stability (crosslinking procedure); the reaction was kept under stirring at 50 °C for 2 h. The black precipitate was collected by magnetic bar and rinsed by water acetone before being dried at 50 °C overnight to produce MC-UR (≈22.37 g, d.w.).

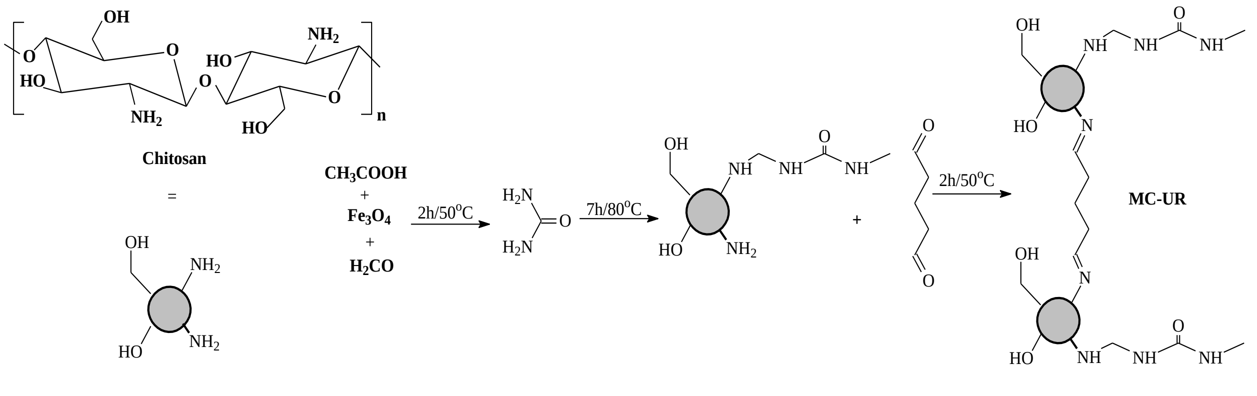

SE.4. Preparation of sulfonating agent (N(SO3Na)3)

A closed system of three-necked flask equipped with mechanical stirrer, dropping funnel and a condenser. The reactor, containing sodium bisulfite (20.2 g into 20 mL demineralized water) was heated in oil bath. Sodium nitrite solution (3.4 g dissolved in 5 mL of distilled water) was added drop wise by the dropping funnel into the reactor. The reaction was performed under agitation at 90 °C for 2.5 h. The produced compound (i.e., the sulfonating agent, $N(SO_3Na)_3$), is used for the preparation of the bi-functional material.

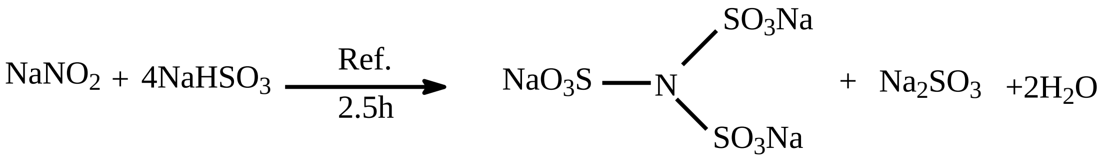

SE.5. Synthesis of sulfonated chitosan urea composite (MC-UR/S)

The prepared composite of the previous step (6 g, d.w.) was added to the sulfonating solution (≈4.5 g). This mixture was refluxed at 85 °C with continuous stirring for 3 h followed by cooling, and collection by magnetic bar for washing with water and ethanol before air-drying at 50 °C for 24 h to yield sulfonated materials (MC-UR/S; ≈ 8.6 g, d.w.).

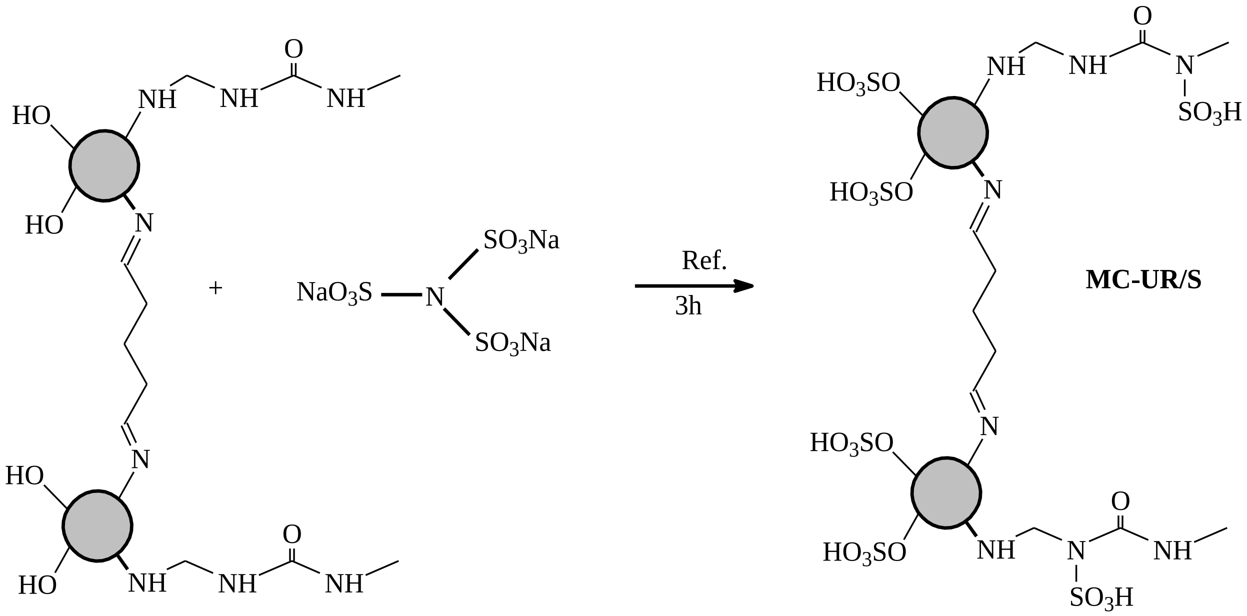

Section SF. Modeling of sorption kinetics and isotherms

Table S12a. Reminder on equations used for modeling uptake kinetics [62, 63].

Model	Equation	Parameters	Ref.
PFORE	$q(t) = q_{eq,1}(1 - e^{-k_1 t})$	$q_{eq,1}$ (mmol g^{-1}): sorption capacity at equilibrium<br>k_1 (min^{-1}): apparent rate constant of PFORE	[62]
PSORE	$q(t) = \frac{q_{eq,2}^2 k_2 t}{1 + k_2 q_{eq,2} t}$	$q_{eq,2}$ (mmol g^{-1}): sorption capacity at equilibrium<br>k_2 (g mmol^{-1} min^{-1}): apparent rate constant of PSORE	[62]
RIDE	$\frac{q(t)}{q_{eq}} = 1 - \sum_{n=1}^{\infty} \frac{6\alpha(\alpha+1)\exp\left(\frac{-D_e q_n^2}{r^2} t\right)}{9 + 9\alpha + q_n^2\alpha^2}$<br>With q_n being the non-zero roots of<br>$\tan q_n = \frac{3\, q_n}{3 + \alpha q_n^2}$ and $\frac{m\, q}{V\, C_0} = \frac{1}{1+\alpha}$	D_e (m^2 min^{-1}) : Effective diffusivity coefficient	[63]

(m (g): mass of sorbent; V (L): volume of solution; C_0 (mmol L^{-1}): initial concentration of the solution).

Table S12b. Reminder on equations used for modeling sorption isotherms.

Model	Equation	Parameters	Ref.
Langmuir	$q_{eq} = \frac{q_{m,L} C_{eq}}{1 + b_L C_{eq}}$	$q_{m,L}$ (mmol g^{-1}): Sorption capacity at saturation of monolayer<br>b_L (L mmol^{-1}): Affinity coefficient	[64]
Freundlich	$q_{eq} = k_F C_{eq}^{1/n_F}$	k_F (mmol g^{-1})/(mmol L^{-1})^{n_F} and n_F: empirical parameters of Freundlich equation	[65]
Sips	$q_{eq} = \frac{q_{m,S} b_S C_{eq}^{1/n_S}}{1 + b_S C_{eq}^{1/n_S}}$	$q_{m,L}$ (mmol g^{-1}), b_S (mmol L^{-1})^{n_S}, and n_S: empirical parameters of Sips equation<br>(based on Langmuir and Freundlich equations)	[66]
Temkin	$q_{eq} = \frac{RT}{b_T} \ln(A_T\, C_{eq})$	A_T (L mmol^{-1}): equilibrium binding capacity;<br>b_T: Temkin constant related to sorption heat (J kg^{-1} mol^{-2})	[67]
D-R*	$q_{eq} = q_{m,DR} exp\left\{-\beta_{DR}\left[RT\, ln\left(1 + \frac{1}{C_{eq}}\right)\right]^2\right\}$	$q_{m,DR}$ (mmol g^{-1}): maximum adsorption capacity;<br>β_{DR} (mol^2 kJ^{-2}): constant associated with adsorption energy.<br>$E_{DR} = \frac{1}{\sqrt{2\beta_{DR}}}$: mean free energy of sorption (kJ mol^{-1})	[68]

, herein Ce must be expressed in molar unit for respecting the dimensionless term ($1/C_{eq}$ = C/Ceq, where C* is the arbitrary concentration

Akaike Information Criterion, AIC [69]:

$$AIC = N \ln\left(\frac{\sum_{i=0}^{N}\left(y_{i,exp.} - y_{i,model}\right)^2}{N}\right) + 2N_p + \frac{2N_p(N_p+1)}{N - N_p - 1}$$

Where N is the number of experimental points, N_p the number of model parameters, $y_{i,exp.}$ and $y_{i,model}$ the experimental and calculated values of the tested variable.

References

1. Zhang, Y.; Hamza, M. F.; Vincent, T.; Roux, J.-C.; Faur, C.; Guibal, E., Tuning the sorption properties of amidoxime-functionalized algal/polyethyleneimine beads for La(III) and Dy(III) using EDTA: Impact of metal speciation on selective separation. *Chem. Eng. J.* **2021**, 133214.
2. Wei, Y.; Salih, K. A. M.; Rabie, K.; Elwakeel, K. Z.; Zayed, Y. E.; Hamza, M. F.; Guibal, E., Development of phosphoryl-functionalized algal-PEI beads for the sorption of Nd(III) and Mo(VI) from aqueous solutions – Application for rare earth recovery from acid leachates. *Chem. Eng. J.* **2021,** 412, 127399.
3. Hamza, M. F.; Salih, K. A. M.; Abdel-Rahman, A. A. H.; Zayed, Y. E.; Wei, Y.; Liang, J.; Guibal, E., Sulfonic-functionalized algal/PEI beads for scandium, cerium and holmium sorption from aqueous solutions (synthetic and industrial samples). *Chem. Eng. J.* **2021,** 403, 126399.
4. Yang, X.; Debeli, D. K.; Shan, G.; Pan, P., Selective adsorption and high recovery of La^{3+} using graphene oxide/poly (N-isopropyl acrylamide-maleic acid) cryogel. *Chem. Eng. J.* **2020,** 379, 122335.
5. Nkinahamira, F.; Alsbaiee, A.; Zeng, Q.; Li, Y.; Zhang, Y.; Feng, M.; Yu, C.-P.; Sun, Q., Selective and fast recovery of rare earth elements from industrial wastewater by porous beta-cyclodextrin and magnetic beta-cyclodextrin polymers. *Water Res.* **2020,** 181, 115857.
6. Guo, Z.; Li, Q.; Li, Z.; Liu, C.; Liu, X.; Liu, Y.; Dong, G.; Lan, T.; Wei, Y., Fabrication of efficient alginate composite beads embedded with N-doped carbon dots and their application for enhanced rare earth elements adsorption from aqueous solutions. *J. Colloid Interface Sci.* **2020,** 562, 224-234.
7. Cao, X.; Wang, Q.; Wang, S.; Man, R., Preparation of a novel polystyrene-poly(hydroxamic acid) copolymer and its adsorption properties for rare earth metal ions. *Polymers* **2020,** 12, (9), 1905.
8. Ahmad, R.; Ali, Z.; Khan, A. A.; Rehman, N. U., Terbium extraction by functionalized surface: experimental and DFT approach. *Adsorption-Journal of the International Adsorption Society* **2020,** 26, (1), 117-125.
9. Zheng, X.; Zhang, Y.; Bian, T.; Zhang, Y.; Zhang, F.; Yan, Y., Selective extraction of gadolinium using free-standing imprinted mesoporous carboxymethyl chitosan films with high capacity. *Cellulose* **2019,** 26, (2), 1209-1219.
10. Callura, J. C.; Perkins, K. M.; Baltrus, J. P.; Washburn, N. R.; Dzombak, D. A.; Karamalidis, A. K., Adsorption kinetics, thermodynamics, and isotherm studies for functionalized lanthanide-chelating resins. *J. Colloid Interface Sci.* **2019,** 557, 465-477.
11. Wang, F.; Zhao, J.; Liu, H.; Luo, Y.; Wang, W., Preparation of double carboxylic corn stalk gels and their adsorption properties towards rare earths(III). *Waste Biomass Valorization* **2018,** 9, (10), 1945-1954.
12. Ravi, S.; Lee, Y.-R.; Yu, K.; Ahn, J.-W.; Ahn, W.-S., Benzene triamido-tetraphosphonic acid immobilized on mesoporous silica for adsorption of Nd^{3+} ions in aqueous solution. *Microporous Mesoporous Mater.* **2018,** 258, 62-71.
13. Hisada, M.; Kawase, Y., Recovery of rare-earth metal neodymium from aqueous solutions by poly-gamma-glutamic acid and its sodium salt as biosorbents: Effects of solution pH on neodymium recovery mechanisms. *J. Rare Earths* **2018,** 36, (5), 528-536.
14. Rahman, M. L.; Biswas, T. K.; Sarkar, S. M.; Yusoff, M. M.; Sarjadi, M. S.; Arshad, S. E.; Musta, B., Adsorption of rare earth metals from water using a kenaf cellulose-based poly(hydroxamic acid) ligand. *J. Mol. Liq.* **2017,** 243, 616-623.
15. Zhao, F.; Repo, E.; Meng, Y.; Wang, X.; Yin, D.; Sillanpaa, M., An EDTA-beta-cyclodextrin material for the adsorption of rare earth elements and its application in preconcentration of rare earth elements in seawater. *J. Colloid Interface Sci.* **2016,** 465, 215-224.
16. Gui, W.; Yang, Y.; Zhu, X., High-efficiency recovery of rare earth ions by hydrolyzed poly(styrene-co-maleic anhydride). *J. Appl. Polym. Sci.* **2016,** 133, (29), 43676.
17. Xu, S.; Wang, Z.; Gao, Y.; Zhang, S.; Wu, K., Adsorption of rare earths(III) using an efficient sodium alginate hydrogel cross-linked with poly-gamma-glutamate. *PLoS One* **2015,** 10, (5), 124826.
18. Galhoum, A. A.; Mahfouz, M. G.; Abdel-Rehem, S. T.; Gomaa, N. A.; Atia, A. A.; Vincent, T.; Guibal, E., Diethylenetriamine-functionalized chitosan magnetic nano-based particles for the sorption of rare earth metal ions Nd(III), Dy(III) and Yb(III). *Cellulose* **2015,** 22, (4), 2589-2605.
19. Galhoum, A. A.; Mafhouz, M. G.; Abdel-Rehem, S. T.; Gomaa, N. A.; Atia, A. A.; Vincent, T.; Guibal, E., Cysteine-functionalized chitosan magnetic nano-based particles for the recovery of light and heavy rare earth metals: uptake kinetics and sorption isotherms. *Nanomaterials* **2015,** 5, (1), 154-179.
20. Ahmadzadeh, M.; Romero, C.; McCloy, J., Magnetic analysis of commercial hematite, magnetite, and their mixtures. *AIP Advances* **2018,** 8, (5), 056807.
21. Ibanescu, A.; Alexandrica, M. C.; Hritcu, D.; Chiscan, O.; Popa, M. I., Magnetite/chitosan composite particles as adsorbents for Reactive Blue 19 dye. *Green Mater.* **2018,** 6, (4), 149-156.
22. Bezdorozhev, O.; Kolodiazhnyi, T.; Vasylkiv, O., Precipitation synthesis and magnetic properties of self-assembled magnetite-chitosan nanostructures. *J. Magn. Magn. Mater.* **2017,** 428, 406-411.
23. Lei, C.; Wen, F.; Chen, J.; Chen, W.; Huang, Y.; Wang, B., Mussel-inspired synthesis of magnetic carboxymethyl chitosan aerogel for removal cationic and anionic dyes from aqueous solution. *Polymer* **2021,** 213, 123316.
24. Cheraghipour, E.; Pakshir, M., Process optimization and modeling of Pb(II) ions adsorption on chitosan-conjugated magnetite nano-biocomposite using response surface methodology. *Chemosphere* **2020,** 260, 127560.

25. Jiang, H.; Liang, J.; Grant, J. T.; Su, S. J.; Bunning, T. J.; Cooper, T. M.; Adams, W. W., Characterization of chitosan and rare-earth-metal-ion doped chitosan films. *Macromol. Chem. Phys.* **1997,** 198, (5), 1561-1578.
26. Lin-Vien, D.; Colthup, N. B.; Fateley, W. G.; Grasselli, J. G., CHAPTER 10 - Compounds Containing $–NH_2$, –NHR, and $–NR_2$ Groups. In *The Handbook of Infrared and Raman Characteristic Frequencies of Organic Molecules*, Lin-Vien, D.; Colthup, N. B.; Fateley, W. G.; Grasselli, J. G., Eds. Academic Press: San Diego, 1991; pp 155-178.
27. Zhang, Z.; Cao, Y. L.; Chen, L. N.; Huang, Z. Y., Preparation of thiol-functionalized cellulose and its application to the removal of Hg(II) from water environment. *Cellul. Chem. Technol.* **2017,** 51, (5-6), 559-567.
28. Lin-Vien, D.; Colthup, N. B.; Fateley, W. G.; Grasselli, J. G., APPENDIX 3 - A Summary of Characteristic Raman and Infrared Frequencies. In *The Handbook of Infrared and Raman Characteristic Frequencies of Organic Molecules*, Lin-Vien, D.; Colthup, N. B.; Fateley, W. G.; Grasselli, J. G., Eds. Academic Press: San Diego, 1991; pp 477-490.
29. Lin-Vien, D.; Colthup, N. B.; Fateley, W. G.; Grasselli, J. G., CHAPTER 14 - Organic Sulfur Compounds. In *The Handbook of Infrared and Raman Characteristic Frequencies of Organic Molecules*, Lin-Vien, D.; Colthup, N. B.; Fateley, W. G.; Grasselli, J. G., Eds. Academic Press: San Diego, 1991; pp 225-250.
30. Lin-Vien, D.; Colthup, N. B.; Fateley, W. G.; Grasselli, J. G., CHAPTER 9 - Compounds Containing the Carbonyl Group. In *The Handbook of Infrared and Raman Characteristic Frequencies of Organic Molecules*, Lin-Vien, D.; Colthup, N. B.; Fateley, W. G.; Grasselli, J. G., Eds. Academic Press: San Diego, 1991; pp 117-154.
31. Dos Santos Menegucci, J.; Santos, M.-K. M. S.; Santos Dias, D. J.; Chaker, J. A.; Sousa, M. H., One-step synthesis of magnetic chitosan for controlled release of 5-hydroxytryptophan. *J. Magn. Magn. Mater.* **2015,** 380, 117-124.
32. Pylypchuk, I. V.; Kolodynska, D.; Gorbyk, P. P., Gd(III) adsorption on the DTPA-functionalized chitosan/magnetite nanocomposites. *Sep. Sci. Technol.* **2018,** 53, (7), 1006-1016.
33. Corazzari, I.; Nistico, R.; Turci, F.; Faga, M. G.; Franzoso, F.; Tabasso, S.; Magnacca, G., Advanced physico-chemical characterization of chitosan by means of TGA coupled on-line with FTIR and GCMS: Thermal degradation and water adsorption capacity. *Polym. Degrad. Stab.* **2015,** 112, 1-9.
34. Ghamami, S.; Anari, S. K.; Bakhshi, M.; Lashgari, A.; Salgado-Morán, G.; Glossman-Mitnik, D., Preparation and characterization of Cerium (III) doped captopril nanoparticles and study of their photoluminescence properties. *Open Chem.* **2016,** 14, (1), 60-64.
35. Coates, J., Interpretation of Infrared Spectra, A Practical Approach. In *Encyclopedia of Analytical Chemistry* John Wiley & Sons, Ltd.: 2006; pp 1-23.
36. Wang, S.; Yu, D., Adsorption of Cd(II), Pb(II), and Ag(I) in aqueous solution on hollow chitosan microspheres. *J. Appl. Polym. Sci.* **2010,** 118, (2), 733-739.
37. Chen, Y. W.; Wang, J. L., Preparation and characterization of magnetic chitosan nanoparticles and its application for Cu(II) removal. *Chem. Eng. J.* **2011,** 168, (1), 286-292.
38. Borai, E. H.; Hamed, M. G.; El-kamash, A. M.; Siyam, T.; El-Sayed, G. O., Synthesis, characterization and application of a modified acrylamide–styrene sulfonate resin and a composite for sorption of some rare earth elements. *New J. Chem.* **2015,** 39, (9), 7409-7420.
39. Stoia, M.; Istratie, R.; Păcurariu, C., Investigation of magnetite nanoparticles stability in air by thermal analysis and FTIR spectroscopy. *J. Therm. Anal. Calorim.* **2016,** 125, (3), 1185-1198.
40. Fu, H.; Yang, Y.; Zhu, R.; Liu, J.; Usman, M.; Chen, Q.; He, H., Superior adsorption of phosphate by ferrihydrite-coated and lanthanum decorated magnetite. *J. Colloid Interface Sci.* **2018,** 530, 704-713.
41. Zheludkevich, M. L.; Tedim, J.; Freire, C. S. R.; Fernandes, S. C. M.; Kallip, S.; Lisenkov, A.; Gandini, A.; Ferreira, M. G. S., Self-healing protective coatings with "green" chitosan based pre-layer reservoir of corrosion inhibitor. *J. Mater. Chem.* **2011,** 21, (13), 4805-4812.
42. Szymanski, H. A., Introduction to Theoretical Infrared Spectroscopy. In *Progress in Infrared Spectroscopy: Volume 1*, Szymanski, H. A., Ed. Springer US: Boston, MA, 1962; pp 1-6.
43. Sorlier, P.; Denuzière, A.; Viton, C.; Domard, A., Relation between the degree of acetylation and the electrostatic properties of chitin and chitosan. *Biomacromolecules* **2001,** 2, (3), 765-772.
44. Hamza, M. F.; Hamad, D. M.; Hamad, N. A.; Abdel-Rahman, A. A. H.; Fouda, A.; Wei, Y.; Guibal, E.; El-Etrawy, A.-A. S., Functionalization of magnetic chitosan microparticles for high-performance removal of chromate from aqueous solutions and tannery effluent. *Chem. Eng. J.* **2022,** 428, 131775.
45. Gustafsson, J. P. *Visual MINTEQ, https://vminteq.lwr.kth.se/, Accessed: April 2019*, ver. 3.1; KTH, Royal Institute of Technology: Stockholm, Sweden, 2013.
46. Dakroury, G. A.; Maree, R. M.; El-Shazly, E. A. A.; Allan, K. F., Synthesize of poly (acrylamide-co-itaconic/TiO_2) nanocomposite for Ce(III) sorption from monazite leachate. *J. Polym. Environ.* **2022,** 30, (5), 1942-1958.
47. Sinha, S.; De, S.; Mishra, D.; Shekhar, S.; Agarwal, A.; Sahu, K. K., Phosphonomethyl iminodiacetic acid functionalized metal organic framework supported PAN composite beads for selective removal of La(III) from wastewater: Adsorptive performance and column separation studies. *J. Hazard. Mater.* **2022,** 425, 127802.
48. Shahnaz, T.; Vishnu Priyan, V.; Jayakumar, A.; Narayanasamy, S., Magnetic nanocellulose from *Cyperus rotundas* grass in the absorptive removal of rare earth element cerium (III): Toxicity studies and interpretation. *Chemosphere* **2022,** 287, 131912.
49. Liu, Z.; Feng, Y.; Li, H., Application of titanium phosphate prepared from acidic titanium dioxide wastewater to remove cerium (III) in aqueous solution. *Colloids Surf., A* **2021,** 630, 127613.

50. Zinicovscaia, I.; Yushin, N.; Humelnicu, D.; Grozdov, D.; Ignat, M.; Demcak, S.; Humelnicu, I., Sorption of Ce(III) by silica SBA-15 and titanosilicate ETS-10 from aqueous solution. *Water* **2021,** 13, (22), 3263.
51. Zhao, L.; Azhar, M. R.; Li, X. J.; Duan, X. G.; Sun, H. Q.; Wang, S. B.; Fang, X. C., Adsorption of cerium (III) by HKUST-1 metal-organic framework from aqueous solution. *J. Colloid Interface Sci.* **2019,** 542, 421-428.
52. Khalil, M.; El-Aryan, Y. F.; El Afifi, E. M., Sorption performance of light rare earth elements using zirconium titanate and polyacrylonitrile zirconium titanate ion exchangers. *Part. Sci. Technol.* **2018,** 36, (5), 618-627.
53. Sadovsky, D.; Brenner, A.; Astrachan, B.; Asaf, B.; Gonen, R., Biosorption potential of cerium ions using Spirulina biomass. *J. Rare Earths* **2016,** 34, (6), 644-652.
54. Zhu, Y.; Wang, W.; Zheng, Y.; Wang, F.; Wang, A., Rapid enrichment of rare-earth metals by carboxymethyl cellulose-based open-cellular hydrogel adsorbent from HIPEs template. *Carbohydr. Polym.* **2016,** 140, 51-58.
55. Chen, T.; Yan, C.; Wang, Y.; Tang, C.; Zhou, S.; Zhao, Y.; Ma, R.; Duan, P., Synthesis of activated carbon-based amino phosphonic acid chelating resin and its adsorption properties for Ce(III) removal. *Environ. Technol.* **2015,** 36, (17), 2168-2176.
56. Torab-Mostaedi, M.; Asadollahzadeh, M.; Hemmati, A.; Khosravi, A., Biosorption of lanthanum and cerium from aqueous solutions by grapefruit peel: equilibrium, kinetic and thermodynamic studies. *Res. Chem. Intermed.* **2013,** 41, (2), 559-573.
57. Torab-Mostaedi, M., Biosorption of lanthanum and cerium from aqueous solutions using tangerine (Citrus reticulate) peel: Equilibrium, kinetic and thermodynamic studies. *Chem. Ind. Chem. Eng. Q.* **2013,** 19, (1), 79-88.
58. Vijayaraghavan, K.; Sathishkumar, M.; Balasubramanian, R., Biosorption of lanthanum, cerium, europium, and ytterbium by a brown marine alga, *Turbinaria conoides*. *Ind. Eng. Chem. Res.* **2010,** 49, (9), 4405-4411.
59. Vijayaraghavan, K.; Balasubramanian, R., Single and binary biosorption of cerium and europium onto crab shell particles. *Chem. Eng. J.* **2010,** 163, (3), 337-343.
60. Sert, Ş.; Kütahyali, C.; İnan, S.; Talip, Z.; Çetinkaya, B.; Eral, M., Biosorption of lanthanum and cerium from aqueous solutions by *Platanus orientalis* leaf powder. *Hydrometallurgy* **2008,** 90, (1), 13-18.
61. Massart, R., Preparation of aqueous magnetic liquids in alkaline and acidic media. *IEEE Trans. Magn.* **1981,** 17, (2), 1247-1249.
62. Ho, Y. S.; McKay, G., Pseudo-second order model for sorption processes. *Process Biochemistry* **1999,** 34, (5), 451-465.
63. Crank, J., *The Mathematics of Diffusion*. 2nd. ed.; Oxford University Press: Oxford, U.K., 1975; p 414.
64. Langmuir, I., The adsorption of gases on plane surfaces of glass, mica and platinum. *J. Amer. Chem. Soc.* **1918,** 40, 1361-1402.
65. Freundlich, H. M. F., Uber die adsorption in lasungen. *Z. Phys. Chem.* **1906,** 57, 385-470.
66. Tien, C., *Adsorption Calculations and Modeling*. Butterworth-Heinemann: Newton, MA, 1994; p 243.
67. Kegl, T.; Kosak, A.; Lobnik, A.; Novak, Z.; Kralj, A. K.; Ban, I., Adsorption of rare earth metals from wastewater by nanomaterials: A review. *J. Hazard. Mater.* **2020,** 386, 121632.
68. Puccia, V.; Avena, M. J., On the use of the Dubinin-Radushkevich equation to distinguish between physical and chemical adsorption at the solid-water interface. *Colloid Interface Sci. Commun.* **2021,** 41, 100376.
69. Falyouna, O.; Eljamal, O.; Maamoun, I.; Tahara, A.; Sugihara, Y., Magnetic zeolite synthesis for efficient removal of cesium in a lab-scale continuous treatment system. *J. Colloid Interface Sci.* **2020,** 571, 66-79.
